# Synchronization of Isolated Downstates (K-Complexes) May Be Caused by Cortically-Induced Disruption of Thalamic Spindling

**DOI:** 10.1371/journal.pcbi.1003855

**Published:** 2014-09-25

**Authors:** Rachel A. Mak-McCully, Stephen R. Deiss, Burke Q. Rosen, Ki-Young Jung, Terrence J. Sejnowski, Hélène Bastuji, Marc Rey, Sydney S. Cash, Maxim Bazhenov, Eric Halgren

**Affiliations:** 1 Department of Neurosciences, University of California, San Diego, San Diego, California, United States of America; 2 Computer Science and Engineering Department, University of California, San Diego, San Diego, California, United States of America; 3 Department of Radiology, University of California, San Diego, San Diego, California, United States of America; 4 Department of Neurology, Seoul National University College of Medicine, Seoul, Korea; 5 Division of Biological Sciences, University of California, San Diego, San Diego, California, United States of America; 6 Howard Hughes Medical Institute, The Salk Institute for Biological Studies, La Jolla, California, United States of America; 7 Central Integration of Pain, Lyon Neuroscience Research Center, INSERM, U1028; CNRS, UMR5292; Université Claude Bernard, Lyon, Bron, France; 8 Unité d'Hypnologie, Service de Neurologie Fonctionnelle et d'Épileptologie, Hôpital Neurologique, Hospices Civils de Lyon, Bron, France; 9 Clinical Neurophysiology Sleep Unit, APHM, Timone Hospital, Aix Marseille Université, Marseille, France; 10 Department of Neurology, Massachusetts General Hospital and Harvard Medical School, Harvard University, Boston, Massachusetts, United States of America; 11 Department of Cell Biology and Neuroscience, University of California, Riverside, Riverside, California, United States of America; 12 Department of Psychiatry, University of California, San Diego, San Diego, California, United States of America; Université Paris Descartes, Centre National de la Recherche Scientifique, France

## Abstract

Sleep spindles and K-complexes (KCs) define stage 2 NREM sleep (N2) in humans. We recently showed that KCs are isolated downstates characterized by widespread cortical silence. We demonstrate here that KCs can be quasi-synchronous across scalp EEG and across much of the cortex using electrocorticography (ECOG) and localized transcortical recordings (bipolar SEEG). We examine the mechanism of synchronous KC production by creating the first conductance based thalamocortical network model of N2 sleep to generate both spontaneous spindles and KCs. Spontaneous KCs are only observed when the model includes diffuse projections from restricted prefrontal areas to the thalamic reticular nucleus (RE), consistent with recent anatomical findings in rhesus monkeys. Modeled KCs begin with a spontaneous focal depolarization of the prefrontal neurons, followed by depolarization of the RE. Surprisingly, the RE depolarization leads to decreased firing due to disrupted spindling, which in turn is due to depolarization-induced inactivation of the low-threshold Ca^2+^ current (I_T_). Further, although the RE inhibits thalamocortical (TC) neurons, decreased RE firing causes decreased TC cell firing, again because of disrupted spindling. The resulting abrupt removal of excitatory input to cortical pyramidal neurons then leads to the downstate. Empirically, KCs may also be evoked by sensory stimuli while maintaining sleep. We reproduce this phenomenon in the model by depolarization of either the RE or the widely-projecting prefrontal neurons. Again, disruption of thalamic spindling plays a key role. Higher levels of RE stimulation also cause downstates, but by directly inhibiting the TC neurons. SEEG recordings from the thalamus and cortex in a single patient demonstrated the model prediction that thalamic spindling significantly decreases before KC onset. In conclusion, we show empirically that KCs can be widespread quasi-synchronous cortical downstates, and demonstrate with the first model of stage 2 NREM sleep a possible mechanism whereby this widespread synchrony may arise.

## Author Summary

EEG in the most common stage of human sleep is dominated by K-complexes (KCs) and sleep spindles (bursts of 10–14 Hz oscillations) occupying the thalamus and cortex. Recently, we discovered that KCs are brief moments when the cortex becomes almost completely silent. Here, using recordings directly from the cortex of epileptic patients, we show that KCs can be quasi-synchronous across widespread cortical areas, and ask what mechanism could produce such a phenomenon. We created the first network model of realistic cortical and thalamic neurons, which spontaneously generate KCs as well as sleep spindles. We showed that the membrane channels in the reticular nucleus of the thalamus can be inactivated by excitatory inputs from the cortex, and this disrupts the spindle-generating network, which can trigger widespread cortical silence. The model prediction that thalamic spindle disruption occurs prior to KC was then observed in simultaneous recordings from the human thalamus and cortex. Understanding the cellular and network mechanisms whereby KCs arise is crucial to understanding its roles in maintaining sleep and consolidating memories.

## Introduction

The EEG during deep non-rapid eye movement sleep (NREM stage N3) is dominated by very large slow oscillations (SO) at ∼1 Hz. Intracellular recordings show that the SO represents alternating ‘upstates’ when neural activity is comparable to waking, and ‘downstates’ when most pyramidal (PY) and interneurons (IN) stop firing and synaptic activity is very low [1]–[3]. The same pattern is observed in humans where the cortical silence during downstates is reflected in a severe drop in high gamma power [4]. The most common sleep stage is N2, a lighter form of NREM sleep, characterized by K-complexes (KC) and sleep spindles. Recently, it was shown that the main component of the KC is the cortical downstate, occurring in relative isolation, i.e., without a preceding upstate [5]. The fact that the KC can be evoked by a weak sensory input, and reflects cortical inactivity, supports a role for the KC as a sleep protective mechanism, suppressing arousal in response to stimuli that are judged by the sleeping brain to be safe [6], [7]. In addition, KC, SO, and sleep spindles have been observed to reflect the hippocampal-cortical interaction during memory replay and may play a critical role orchestrating the consolidation of temporary episodic memories into the permanent semantic store [8], [9].

SOs are ubiquitous in mammalian sleep, allowing their neural substrate to be well-delineated. The occurrence of SOs in the undercut cortex [10] and in cortical slices [11], [12], suggests that intracortical mechanisms are critical for SO generation. During the SO, possible mechanisms for the downstates that follow upstates include synaptic fatigue, active K^+^ currents, or synaptic inhibition [2], [11], [13]–[16]. However, these mechanisms inadequately explain the KC origin because they depend on a preceding upstate which typically does not occur before the KC. In these mechanisms, the downstates of the SO not only arise locally as a consequence of the upstate, but it is the upstate which spreads and the spread of the downstate is again dependent on a preceding upstate which is not present before the KC. This spread of SO upstates is modeled as carried by local excitatory cortico-cortical connections, and thus comprise travelling waves. Travelling waves have been observed in an in vitro model of SO [14], and have been inferred to travel from prefrontal to posterior cortex based on scalp EEG during SO [17]. Since the KC is identical to an isolated component of the SO, it has often been assumed that it also is a cortico-cortical travelling wave. On the other hand, qualitative descriptions of KCs in EEG suggest that they appear quasi-synchronously across the scalp [6], [7]. Since this issue is crucial for constraining mechanistic models of the KC, we first examined it quantitatively before constructing our model. Using scalp EEG, we confirmed earlier reports that KCs can appear quasi-synchronously across frontal and posterior sites. However, the scalp KC is comprised of multiple overlapping EEG components whose overlap could affect their apparent latencies. Thus, we confirmed the EEG findings with widespread recordings directly from the pial surface (ECOG) as well as recordings from electrode pairs that span the cortex (bipolar SEEG). Although KCs were not absolutely synchronous across the cortical surface, their relative latencies between distant locations were too short to be consistent with current models of cortical travelling waves, and we therefore define them as quasi-synchronous.

In addition to KCs, N2 is characterized by sleep spindles, which are waxing and waning bursts of 10–14 Hz oscillations, each lasting ∼1 s. Like KCs, scalp EEG also suggests that sleep spindles are widely synchronous, but simultaneous magnetoencephalographic and intracranial recordings have demonstrated multiple asynchronous generators [18]–[20]. Unlike KCs, spindles can be generated by the isolated thalamus [21] through the interaction of GABAergic thalamic reticular nucleus (RE) neurons and excitatory thalamocortical (TC) neurons, reinforced by intrinsic currents [22]. Burst firing of RE neurons hyperpolarizes TC neurons, deinactivating I_T_, and inducing rebound burst firing that excites RE neurons, triggering another cycle. Although spindles and KCs/SOs can thus be generated by the isolated thalamus or cortex, respectively, the close anatomical and physiological integration of these structures suggests that both are involved in generating, and especially synchronizing, both phenomena [21], [23]–[30].

In sum, recent evidence has shown that KCs are isolated states of profound thalamocortical silence, which can appear quasi-synchronously across both hemispheres and multiple lobes. What mechanism could produce such a phenomenon? As we noted above, current models of the SO are not sufficient because the downstates are triggered by preceding upstates in these models, but KCs are not preceded by upstates. Further, these models do not predict that downstates will be synchronous, but that they will spread like a wave across the cortical surface. These models also do not include the thalamus (thus preventing the potential synchronizing effects of the thalamocortical interactions to be assessed), and they do not include spindles, which commonly occur in close proximity to KCs during N2. Finally, given the crucial roles that the H and T currents play in generating thalamocortical sleep rhythms, it is essential that they be included in models for these phenomena. Here we describe the first such model, including both the thalamus and cortex, which produces spindles, as well as spontaneous and evoked KCs. In the model, the same limited number of RE, TC, PY, and IN cells are interconnected in local and distant feedback and feed forward loops, as is typically observed in vivo [2]. However, in some simulations we also included connections from a limited set of PY neurons to all of the RE neurons, reflecting the recent anatomical finding in rhesus monkeys, that PY neurons in areas 46, 13, and 9 are unique in projecting widely to the RE neurons [31]. Activation of these PY neurons would widely excite RE neurons, thus inhibiting TC neurons; the sudden removal of tonic TC drive would trigger a widespread synchronized cortical KC.

Using a detailed conductance based thalamocortical network model of N2, we find that, indeed, these widespread RE connections from a limited PY region are necessary for spontaneous KCs to occur. Furthermore, applied depolarization of this focal prefrontal area does lead to a KC, as predicted, but it is via an unexpected mechanism: RE depolarization inactivates I_T_, thus disrupting thalamic spindles, which removes tonic thalamocortical excitation. In order to test the robustness of the model's predictions for empirical data, we analyze rare human recordings made simultaneously from the cortex and thalamus during NREM sleep and find that a drop in thalamic spindling precedes the prefrontal KC. In summary, this paper presents the first comprehensive computational neural model of stage N2 sleep, and demonstrates, using a combination of modeling and empirical observations, a possible mechanism whereby an interaction of network and channel properties results in quasi-synchronous cortical downstates (KCs), triggered by the interruption of thalamic spindling.

## Results

First, we present evidence from scalp EEG and direct cortical recordings that help place constraints on the model. Scalp EEG demonstrates that KCs can be quasi-synchronous over the scalp and direct cortical recordings show that evoked and spontaneous KCs can be quasi-synchronous over much of the cortical surface in humans. Second, we describe the KCs produced spontaneously by the thalamocortical model when the cortex contains neurons which project diffusely to RE, and how the rate of KCs depends on the strength and number of such connections. The mechanism whereby KCs arose was studied in the model by examining different intracellular and network parameters associated with the spontaneous KCs, as well as those associated with KCs evoked by direct depolarization of RE neurons or by stimulating the cortical neurons projecting diffusely to RE. The model KCs resulted from removal of cortical excitation when TC neurons stopped firing. Although this can result from inhibitory input from RE neurons that are strongly stimulated, in most cases direct or indirectly-induced depolarization of RE neurons resulted in decreased firing of both RE and TC neurons by disruption of spindling, a consequence of inactivation of I_T_. Third, as an empirical test of the model's predictions, we report a single patient with simultaneous direct thalamic and cortical recordings showing spindling in the human thalamus that is disrupted before the KC.

### Empirical Measures

#### KCs measured with scalp EEG do not always show a systematic propagation from anterior to posterior channels

**Figure 1:**
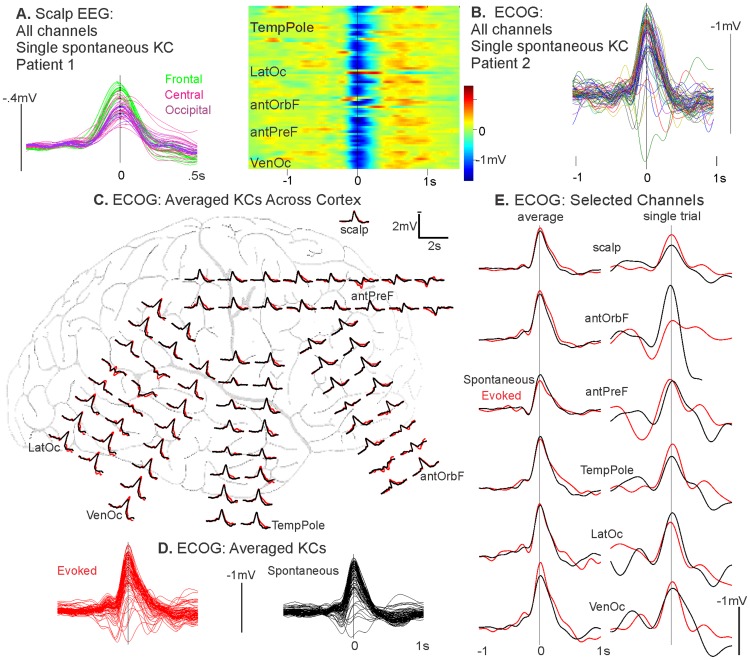
Quasi-synchrony and topography of spontaneous and evoked KCs in EEG and ECOG. A, An individual spontaneous KC shows quasi-synchrony over frontal, central, and occipital scalp EEG channels. Overlaid channels participating in the KC are color-coded by scalp position: frontal channels in green, central in pink, and occipital in purple. There is no evidence of a systematic propagation of green frontal to purple posterior channels. The vertical black line is plotted at the time point where there is a 0 ms delay in the timing of the most negative peak of the KC in channels across the anterior to posterior axis: Fp1, Fpz, F1, Fc2, C4, Po3, and Po8. B, Almost all ECOG channels show a quasi-synchronous surface negative potential at the time of a single spontaneous KC detected at the scalp. The left plot depicts the color coded local field potentials while the right plot depicts the waveforms of this KC. Regions listed are: temporal pole (TempPole), lateral occipital (LatOc), anterior orbitofrontal (antOrbF), anterior prefrontal (antPreF), and ventral occipital (VenOc). C, Both spontaneous (black) and evoked (red) KCs in ECOG are characterized by a quasi-synchronous and widespread surface negativity. KCs were averaged over trials on the most negative peak of the KC as detected at the pictured scalp EEG electrode, C4. Waveforms are the average of 86 spontaneous (black) and 42 evoked (red) KCs. Patient is the same as in B. D, Traces of KC averages from all channels pictured in C are superimposed to demonstrate the regularity of the waveforms across sites. E, On both an average and single trial basis, spontaneous and evoked KCs show similar topographical profiles that are quasi-synchronous across the cortex. The scalp EEG electrode and ECOG channels from across the cortex (selected to be maximally far apart) are pictured for the averaged spontaneous or evoked KC, as depicted in C & D, as well as a single trial. The vertical black line indicates time zero of the most negative peak of the scalp KC on the average (left) and the single trial (right). Channel location regions listed are the same as in B.

The prevailing view in the field is that slow oscillations propagate systematically in an anterior to posterior direction via a cortico-cortical mechanism, and it has often been assumed that KCs travel in the same manner. We demonstrate here with a spontaneous KC measured using scalp EEG that this systematic front-to-back propagation is not the rule for all KCs (Figure 1A). Figure 1A shows the overlay of all scalp EEG channels participating in the KC from a healthy control subject. KC waveforms are color-coded to indicate their frontal (green), central (pink), or occipital (purple) positions. There is no evidence of a systematic propagation from anterior to posterior, as would be visualized as a progression from green to pink to purple channels. Furthermore, the vertical black line marks the time point where the following channels, which span the anterior to posterior axis, appear absolutely synchronous (i.e. a 0 ms delay in the timing of the most negative peak of the KC, at a resolution of 1.7 ms): Fp1, Fpz, F1, Fc2, C4, Po3, and Po8. The average latency delay of the most negative peak of the KC in frontal channels compared to the most negative peak of the KC in occipital channels is ∼8 ms. As the signal from scalp EEG is smeared by the skull, and latencies of individual components can be shifted by overlapping components, we do not undertake a systematic investigation into the travelling versus quasi-synchronous KCs as measured in scalp EEG. Rather, we quantitatively investigate the existence of quasi-synchronous KCs using direct cortical recordings, as outlined below.

#### Evoked and spontaneous KCs are downstates occurring quasi-synchronously across much of the cortex

Intracranial recordings were obtained from patients undergoing evaluation for their epileptogenic focus: Patient 1 had ECOG grid electrodes on the pial surface, and Patients 2 to 4 had exclusively cortical SEEG. We examined KCs using both of these recording techniques and found that KCs are: quasi-synchronous, widespread across the cortex, and that spontaneous and evoked have similar morphologies and topographies.

We compared the distribution and synchrony of evoked and spontaneous KCs across the cortex using ECOG in Patient 1 (Figure 1B–E). In the single spontaneous KC shown in figure 1B, almost all ECOG channels show a near-synchronous negativity at the time of a KC chosen on the C4 scalp electrode. This widespread and tightly synchronized negativity is apparent in both the time-voltage plot (where each line represents color coded voltage across time of a single channel Figure 1B, left), and the superimposed KC waveforms across all channels (Figure 1B, right).

Figure 1C compares the distribution and synchrony of averaged spontaneous (black waveforms, n = 86) and evoked (red waveforms, n = 42) KCs across the cortex using ECOG grid electrodes from the same patient. Of 54 tones delivered, 42 (∼78%) resulted in an evoked KC. Both spontaneous and evoked KCs were detected on C4 and averaged on the most negative peak of this scalp channel. Both evoked and spontaneous KCs are characterized by a quasi-synchronous surface negativity that is widespread across the cortical surface (Figure 1C). Superimposed averaged traces from all channels demonstrate the similar topography of both types of KCs, implying they have the same generators (Figure 1D). This was tested statistically by calculating the correlation coefficient in the peak amplitudes between spontaneous and evoked KCs across the 71 channels: R was found to be 0.94. In Figure 1E, the scalp EEG channel and ECOG channels selected to represent the most distant relative locations across the cortex are enlarged to depict the similar and quasi-synchronous spontaneous and evoked morphologies that occur for both averaged and single trial KCs. The vertical black line demarcates the KC peak in the scalp EEG channel. Relative to this time, the peak latencies of the displayed channels for the spontaneous (black) KC peaks ranged from −15.6 ms to 7.8 ms (mean 0 ms) for the average, and from −7.8 ms to 7.8 ms (mean 3.1 ms) for the single trial. In particular, the latency difference between the anterior prefrontal channel (antPreF) and the lateral occipital (LatOc), which represent the most distant anterior-posterior sites of the selected channels, was 3.9 ms on average and 0 ms on a single trial basis. The peak latencies for the evoked (red) KC peaks ranged from −7.8 ms to 7.8 ms (mean −0.8 ms) for the average, and from −11.7 ms to 11.7 ms (mean 1.6 ms) for the single trial. For the evoked KCs, the latency difference between antPreF and LatOc was also 3.9 ms for the average and 0 ms for a single trial. The quasi-synchrony of the average ECOG peaks demonstrates that KCs do not show a regular delay between prefrontal and posterior sites that is comparable to that which has been reported for the SO in the scalp EEG (∼120 ms, [17]). Since the quasi-synchrony can also be observed in single trials, the lack of large latency differences in the average peaks is unlikely to be due to KCs slowly propagating on each trial, but in different directions, which would thus be masked in the average.

As is evidenced by the large differences between adjacent ECOG contacts, ECOG is thought to mainly reflect the immediately underlying cortex. However, it is subject to reference effects as well as volume conduction [32]. In order to obtain unambiguously focal cortical recordings, we examined SEEG depth electrode trajectories to identify successive contacts on the same probe, with one above the cortical gray matter in the CSF, and the other just below it in the white matter. Often the external electrode was in the subarachnoid space just lateral to the cortical surface, but in some cases it was medial to midline cortex (i.e., just lateral to the falx cerebri), or within a vertically-trending sulcus. We calculated the transcortical local field potential (LFP) by subtracting the SEEG recorded by the electrode below the gray matter from that recorded by the electrode above it. These contacts were selected to measure transcortical LFP in order to have a very focal recording of local activity.

**Figure 2:**
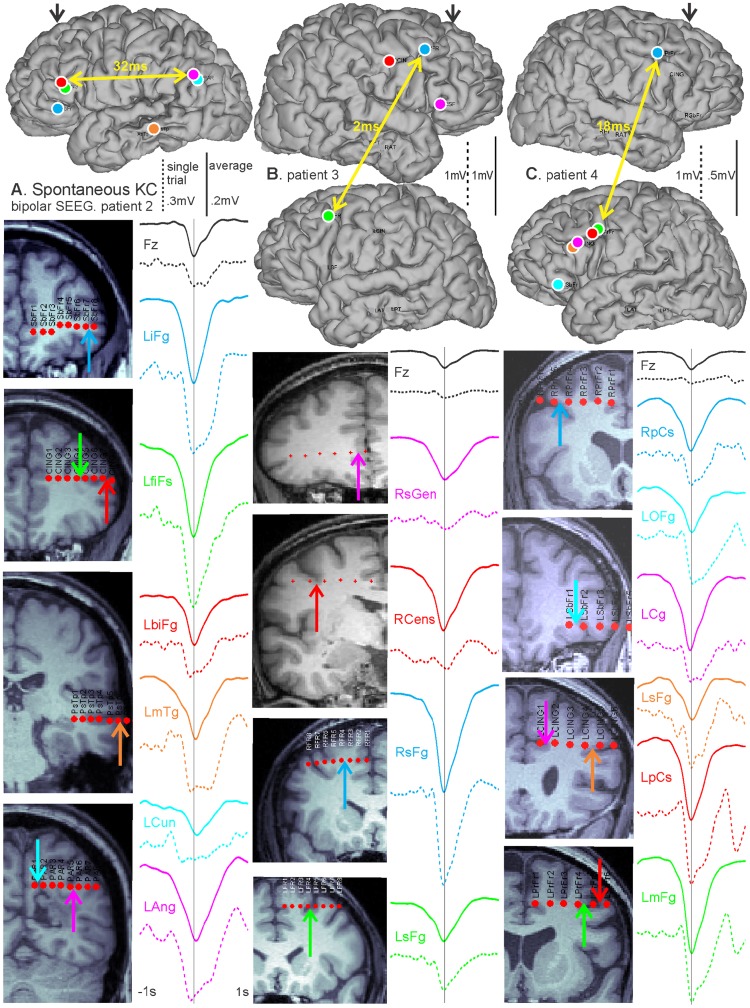
Bipolar SEEG recordings illustrate widespread spontaneous KC quasi-synchrony across lobes and hemispheres. Single spontaneous (dashed lines) and averaged (solid lines) KCs detected on Fz for each patient and computed for each selected pair of bipolar contacts. The color of each waveform corresponds to the colored arrow indicating its location on the reconstructed hemispheres and coronal MRI sections for each patient. A, A single trial and the average of 70 KCs for Patient 2 with contacts across the frontal, temporal, and parietal lobes. On average, there is a 32 ms delay between the anterior (inferior frontal sulcus, LbiFg) and posterior (angular gyrus, LAng) channels shown by the yellow arrows. B, A single trial and the average of 129 KCs for Patient 3 with contacts in both hemispheres. The yellow arrow marks mirrored locations in the left and right superior frontal gyrus (RsFg and LsFg), which have an average KC peak latency delay of 2 ms. C, A single trial and the average of 172 KCs for Patient 4 with contacts in both hemispheres for subfrontal and frontal regions. In mirrored sites in the left and right precentral sulcus (LpCs and RpCs), highlighted by the yellow arrow, there is an average 18 ms peak latency difference.

In Figure 2, spontaneous KCs were analyzed using exclusively cortical bipolar SEEG recordings in three patients, with analyzed channels chosen as described above. For all three patients, KCs were detected on Fz. For each patient, a single spontaneous KC (dashed lines) is displayed with the average KC (solid lines) chosen on Fz and computed for each selected pair of bipolar contacts across the cortex (Figure 2). For each patient, the postoperative CT with the electrodes in place was superimposed on the preoperative MRI to estimate the entry points of SEEG probes on the reconstructed cortical surface, as well as the locations of individual contacts on coronal T1-weighted sections [33]. For each bipolar SEEG recording, the same color is used to denote corresponding waveforms, entry-points, contacts, and anatomical labels. Patient 2 in Figure 2A had electrodes implanted unilaterally in the dorsal and ventral prefrontal, temporal, and parietal cortices. Over the average of 70 KCs, there was a 32 ms peak latency difference between the bank of the inferior frontal sulcus (LbiFg) and the angular gyrus (LAng) (marked by the yellow arrows), which was reduced to 12 ms on a single trial example. Patient 3 in Figure 2B shows a single example KC and an average of 129 KCs that are quasi-synchronous between the hemispheres. Mirrored sites in the left and right superior frontal gyrus (RsFg and LsFg) have a 2 ms latency difference in their averaged KC peaks, marked by the yellow arrows, and a 8 ms single trial KC peak latency difference. Patient 4 in Figure 2C demonstrates the quasi-synchrony of KCs across multiple dorsal and ventral prefrontal and anterior cingulate areas in one hemisphere, as well as between the frontal lobes. On the average of 172 KCs, mirrored sites in the left and right precentral sulcus (LpCs and RpCs) showed a 18 ms peak latency difference (marked by yellow arrows), which was 14 ms in the single trial example. As with the ECOG recording, we include single as well as averaged KCs to demonstrate that quasi-synchrony in the average is not due to travelling waves propagating systematically in different directions on different trials.

#### Quasi-synchronous versus travelling waves

The primary goal of the initial empirical studies was to constrain the important characteristics of KCs that would need to be reproduced in an adequate mechanistic computational model of the KC. Specifically, we sought to determine if the spread of KCs can be accommodated within the range of conduction velocities previously demonstrated in vivo, in vitro, or in modeling to be supported by short and medium range cortico-cortical fibers. We did this by comparing the estimated conduction velocities observed for the KCs in our recordings to those reported for cortical travelling waves found in the mammalian neocortex during sensory processing. These range from 0.1–0.4 m/s [34], up to 0.6 m/s [35]. These studies focus on active visual processing and thus may not be directly relevant to propagation of the KC. For this reason, we also specifically refer to the speeds observed by Massimini *et al.* [17], which is the key study asserting that the slow oscillation is a travelling wave, and to the references cited by that study from the animal and modeling literature. Different comparisons are made for speeds that are measured on the cortical versus the scalp surface. In the scalp EEG recordings presented by Massimini *et al.*, propagation speed was estimated between 1.2 and 7 m/s (mean = 2.5 m/s), with an approximate 120 ms delay from frontal to occipital EEG recording sites [17]. Source modeling by the same group estimated cortical propagation speeds as approximately 2.2 m/s [36]. In cortical recordings from ferret slices, the propagation speed was measured at 0.011 m/s [12]. In a realistic neural model inspired by the latter study, the propagation speed was calculated from 0.003 to 0.008 m/s [14].

We measured the average delay between anterior prefrontal and occipital sites, and found it to be about 8 ms in scalp EEG (Figure 1A), and 25 ms in intracranial recordings, averaged from the intracranial subjects (Figures 1E and 2A). Assuming a distance on the scalp of about 24 cm, and on the cortical surface of about 48 cm (both are underestimates), then this corresponds to a velocity of 30 m/s at the scalp and 20 m/s on the cortical surface. This is ∼30–200 times faster than those observed in cortical travelling waves [34], [35]. It is also at least 10 times faster than that described by Massimini *et al.* [17] for scalp recordings, ∼2,000 times faster than the in vitro study [12] and ∼4,000 times faster than the modeling study [14] that they quote. Thus, our observed average delays between KCs recorded at distant sites are not consistent with the interpretation of KCs as cortical travelling waves. Our observations also fail to support a consistent or obligatory origin of KC in prefrontal cortex. Rather, our results indicate that KCs appear in distant cortical locations with delays that are inconsistent with propagation by short- or medium-range cortico-cortical connections. Since long range cortico-cortical connections are thought to be excitatory as well as sparse, we explored a possible role for the thalamus in synchronizing KC onset.

### Model Simulation Results

The cardinal features identifying human N2 are the occurrence of spindles and KCs, and thus any model of this state must produce both of these phenomena. As demonstrated above using empirical recordings, the model must also produce both spontaneous and evoked KCs that occur quasi-synchronously across the cortex. We used a conductance based thalamocortical model (see Materials and Methods section) composed of 100 pyramidal (PY) neurons, 25 inhibitory interneurons (IN), 50 thalamic reticular (RE) neurons, and 50 thalamocortical (TC) neurons. Our initial model was based on that described by [2] to produce SOs. We modified this model by decreasing the strength of the PY to PY AMPA synapses from 0.15 µS to 0.09 µS. In a very similar model, it has recently been shown that decreasing these intracortical connections causes a transition from N3 to N2 [37]. In our model, decreasing AMPA synaptic strength effectively decreased the relative strength of cortico-cortical, as compared to cortico-thalamo-cortical connections, with the result that spontaneous SOs were eliminated while allowing spontaneous spindles to emerge. However, this model was still inadequate because it did not generate spontaneous KCs.

#### KCs are produced spontaneously

**Figure 3:**
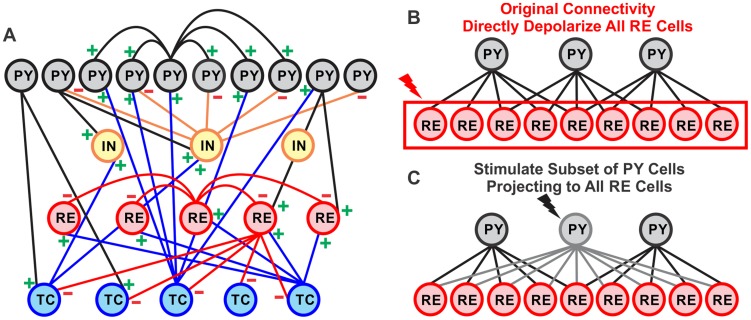
Network geometry and altered projections of thalamocortical model. A, The network contained 100 PY, 25 IN, 50 TC, and 50 RE neurons. The number of projections between PY and RE neurons was altered to test two ways of evoking KCs. B, In the original network geometry, each PY neuron projected to 5 RE neurons. To evoke KCs, all RE neurons were depolarized. C, In the altered connectivity, a subset of PY neurons projected to all RE neurons, while the remaining PY neurons projected to 5 RE neurons. To evoke KCs, only the PY neurons projecting to all RE neurons were stimulated.

Inspired by recent anatomical work demonstrating widespread connections from restricted prefrontal locations to the RE [31], we modified our model to include a fraction of PY neurons (15 PY neurons in these simulations) which project to all RE neurons (see Figure 3C). This modification resulted in spontaneously occurring KCs, as well as spontaneous spindles, thus faithfully reproducing the essential features of N2.

**Figure 4:**
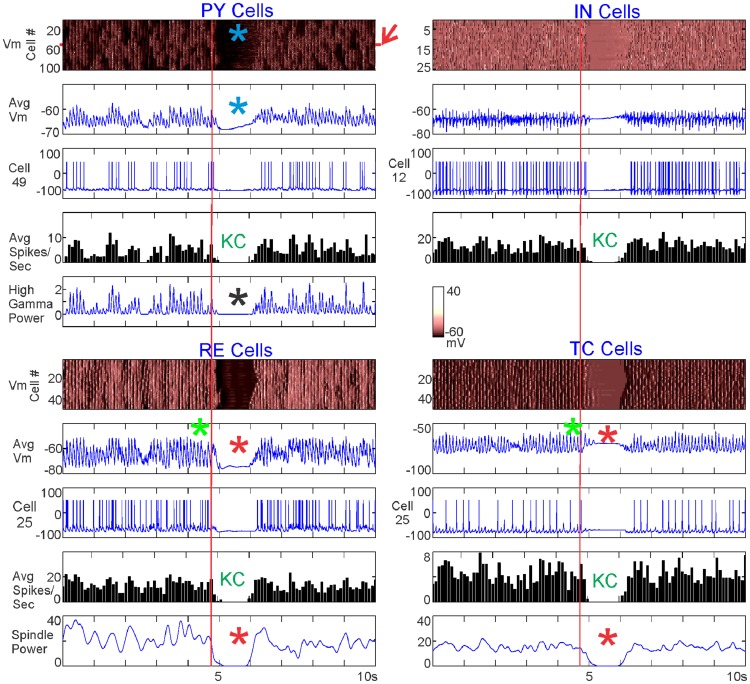
Characteristics of a single spontaneous KC. A KC generated when 15 PY neurons projected to all RE neurons. For each of the neuronal populations, the membrane potential of the individual neurons (Vm), the average membrane potential (Avg Vm), the spiking of a single neuron (Cell), and the average spiking rate (Avg Spikes/Sec) are pictured. In addition, high gamma power is plotted for PY neurons and spindle power is plotted for RE and TC neurons. The KC is characterized by a cessation of firing by all cell types, with a drop in membrane potential (blue asterisks), and high gamma power (black asterisk) in PY neurons. The model exhibited spindling (green asterisks), which dropped in the RE and TC neurons during the cortical KC (red asterisks). The red arrow indicates the 15 PY neurons that are connected to all RE neurons, and the red vertical lines mark the start of RE spindle disruption. The membrane potential color scale displayed in the middle of the figure is the same for all cell populations.

Figure 4 plots an example spontaneous KC produced by the model. For each of the four neuronal populations in the model (PY, IN, TC, RE), we measured the individual membrane potential of each neuron in the population (Figure 4, top plot for each population). For each population, we calculated the averaged membrane potential and the averaged spiking rate. Average membrane potential was calculated by averaging over each neuron's membrane potential and applying a −50 mv spiking threshold to approximate the local field potential (LFP) (Figure 4, second plot for each population). For each population, the spiking pattern of a representative neuron is plotted, with the y-axis specifying the number of that neuron in the population (Figure 4, third plot for each population). The average spiking rate over each population (Figure 4, fourth plot for each population) was calculated over a 100 ms bin by counting each time a neuron's membrane potential reached above −20 mv and dropped below −30 mv the following millisecond. High gamma power (HGP, 70–200 Hz) was calculated for the PY neurons and spindle power (8–13 Hz) was calculated for the TC and RE neurons using Morlet wavelets. We did not distinguish between slow and fast spindles because this is a level of detail that would require a larger model; rather, we observed that spindles occurred in the model at a rate of 8–13 Hz and used this as our bounds for quantifying the strength and time-course of spindles. For both HGP and spindle power, power was calculated for individual neuronal membrane potentials and then averaged.

In Figure 4, blue asterisks and “KC” indicate a spontaneously occurring KC, characterized by a sustained drop in membrane potential and spiking across all populations. A drop in HGP in the PY neurons further confirmed the event as a KC (black asterisk). Spindling in the average membrane potential of TC and RE neurons is marked by green asterisks and shows a clear and sustained drop at or before the time of the KC, along with a drop in spindle power (red vertical lines and red asterisks).

**Figure 5:**
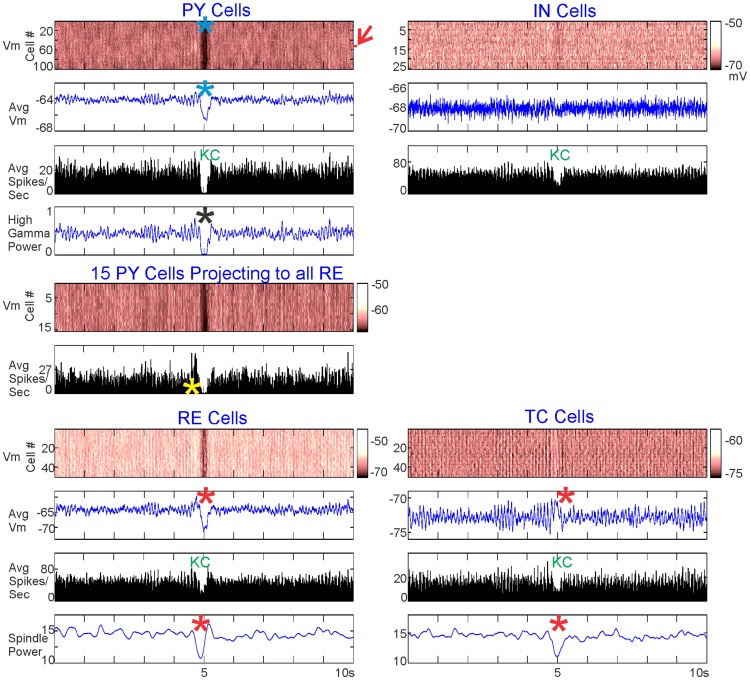
Characteristics of 39 averaged spontaneous KCs. KCs generated when 15 PY neurons projected to all RE neurons, as in Figure 4. For each of the populations, the membrane potential of the individual neurons (Vm), the average membrane potential (Avg Vm), and the average spiking rate (Avg Spikes/Sec) (averaged over the 39 KCs) are pictured. High gamma power is plotted for all PY neurons. In addition, the membrane potential (Vm) and the averaged spiking (Avg Vm) of the 15 PY neurons projecting to all RE neurons, averaged over the 39 KCs, are plotted separately. The red arrow indicates the 15 PY neurons that are connected to all RE neurons. The color scale of the PY membrane potential is the same for all PY neurons and these 15 PY neurons. The averaged KC exhibited the same characteristics as the individual KC shown in Figure 4: cessation of firing by all cell types, and a drop in all PY neurons in membrane potential (blue asterisks), and high gamma power (black asterisk). Spindling also dramatically decreased in the RE and TC neurons during the cortical KC (red asterisks). The 15 PY neurons showed a marked increase in spontaneous firing before the KC (yellow asterisk).

We investigated whether, on average, spontaneous KCs exhibit the same characteristic as the individual spontaneous KC pictured in Figure 4: a drop in TC and RE spindling power at the time of the KC. Figure 5 shows the average of 39 spontaneous KCs generated over 200 seconds of the same simulation described in Figure 4, with 15 PY neurons projecting to all RE neurons. The 39 KCs were chosen based on the PY firing rate criteria for a KC: less than 10 spikes per 100 ms, for at least 200 ms. As expected from these criteria, and as seen in the individual KC, these KCs were characterized by a drop in PY membrane potential and decreased firing in all populations (blue asterisks and “KC,” respectively). In addition, the averaged KCs also showed a drop in HGP in PY (black asterisk) and a disruption of spindling in RE and TC neurons (red asterisks). A red arrow indicates the 15 PY neurons that project to all RE neurons, whose membrane potential and spiking are plotted separately. The firing of these neurons increases before the KC (yellow asterisk).

#### The rate of spontaneous KCs depends on the number of PY neurons projecting to all RE neurons

**Figure 6:**
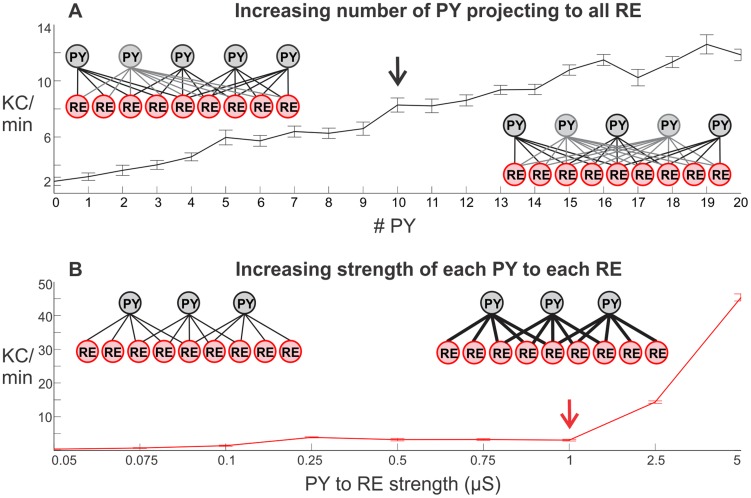
Parametric modulation of spontaneous KC frequency by changing the projection from PY to RE. A, Increasing the number of PY neurons projecting to all RE neurons increased the number of spontaneous KCs in a near linear fashion. B, In contrast, increasing the strength of projections from PY neurons to RE neurons had little effect until a threshold strength was reached and the system began oscillating almost continuously (i.e., generated SOs rather than KCs). In A, the PY to RE configuration schematic on the left illustrates one PY cell projecting to all RE neurons, while each of the remaining PY neurons maintain a projection to 5 RE neurons (as in Figure 3C). The schematic on the right shows how the projection pattern changes with the addition of a second PY neuron projecting to all RE neurons. In B, the schematic on the left illustrates the original model configuration (as in Figure 3B), while the right schematic shows the increasing strength of PY to RE connections. For each value on the x-axis, 10 simulations of 200 s with different random seeds were run. The number of spontaneous KCs was calculated for each run; each value represents the average number of KCs per minute in the 10 runs ± SEM. The black arrow marks 10 PY neurons projecting to all RE neurons while the red arrow marks the original connectivity with a strength of 1 µS between PY and RE neurons.

To better understand the mechanism underlying the generation of spontaneous KCs, we tested which model parameters affected the rate at which KCs are generated. When a subset of PY neurons projected to all RE neurons, increasing the number of such PY neurons in the model increased the number of KCs in a near-linear fashion (Figure 6A). In doing so, however, the overall input strength from the cortex to thalamus was increased. To test if the increase in spontaneous KCs was due to the changed connectivity versus the increased strength, we systematically tested the effects of gradually increasing the strength of each PY neuron projecting to RE neurons in the model, while maintaining the original connectivity where each PY neuron projects to 5 RE neurons. This increased connection strength had little or no effect on the rate of spontaneous KC production until 2.5 or 5 µS, a level corresponding to 5 to 10 times the baseline connection strength of 0.5 µS (Figure 6B). The total PY-RE strength in the baseline model is 500 synapses (100 PY – 5 RE) times 0.5, or 250 µS. Adding 10 PY neurons which project to all RE neurons (Figure 6A, black arrow) produces a total synaptic strength of 475 µS, less than that for the model with the original connectivity when each synapse is set to 1 µS (Figure 6B, red arrow), which totals 500 µS. However, the former simulation resulted in ∼8 KCs per minute (black arrow) whereas the latter resulted in baseline levels (red arrow). Eventually, increasing the strength of all PY-RE synapses with the original connectivity did result in the sharp increase of downstate production. However, these did not appear as isolated downstates (i.e., as KCs) but rather as continuous SOs (0.25–1 Hz) (i.e., resembling N3 rather than N2) [2]. These simulations suggest that widely projecting connections from PY to RE neurons can effectively transform a model that generates spindles to one that also generates spontaneous KCs.

Note that the baseline rate of ∼3–4 KCs/min produced spontaneously by the model is close to rates reported in the literature: ∼2 KCs/min (slightly higher for young subjects and slightly lower for older subjects) [38]; 1.3 KCs/min at baseline and 2.6 KCs/min for recovery sleep [39]; and a 5–10 second evoked KC refractory period (which is equivalent to ∼3–6 KCs/min if assuming a 50% rate of evoking KCs with stimuli delivered at a rate with this refractory period in mind) [40].

#### KCs are evoked either by directly depolarizing all RE neurons or by stimulating a subset of PY neurons projecting to all RE neurons

**Figure 7:**
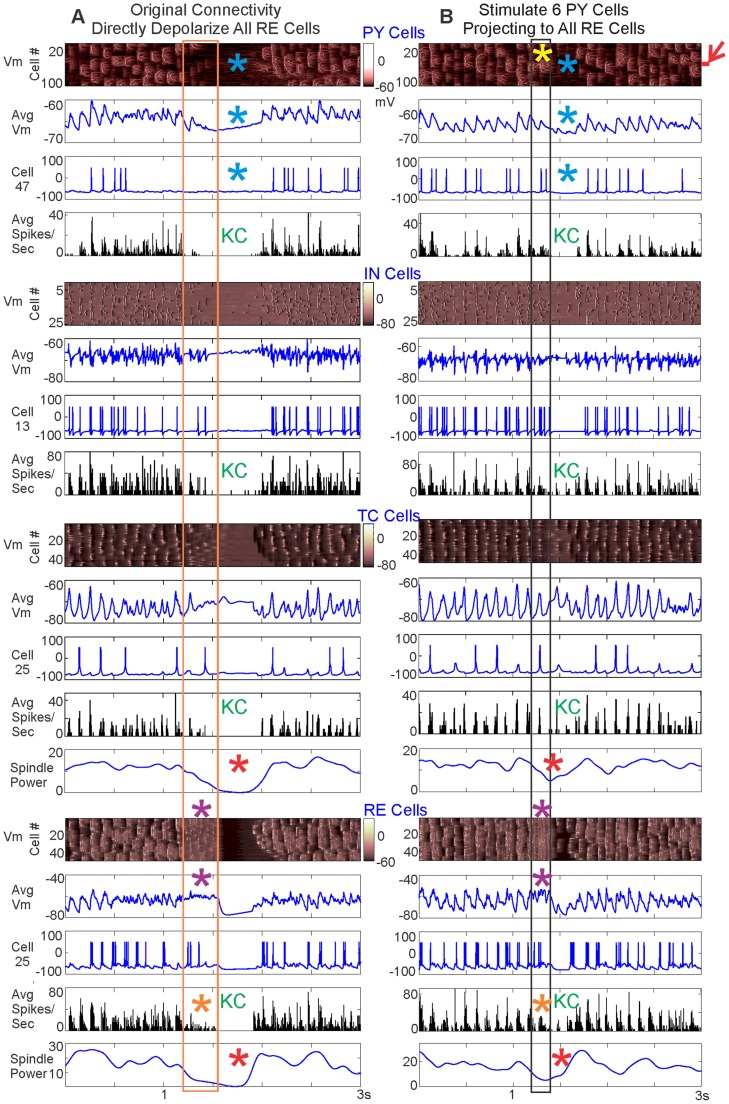
Evoking KCs by directly versus indirectly depolarizing RE. A, KCs are evoked by directly depolarizing all RE neurons for 350 ms at 85.8pA. B, KCs are evoked by stimulating 6 PY neurons, indicated by the red arrow, for 200 ms at 15pA. In A & B, the membrane potential of individual neurons (Vm), the average membrane potential (Avg Vm), the spiking of a single cell, and the average spiking rate (Avg Spikes/Sec) are graphed for each population. The membrane potential color scales are the same for both panels for each cell population. The number of the individual cell graphed is also the same between panels. The length of the RE depolarization is outlined by the orange box and the length of the stimulation of the 6 PY neurons is outlined by the black box. In both cases, the KC was quantified by a drop in the PY membrane potential and the spiking in cell populations (blue asterisks and “KC”). There was a marked increase in the membrane potential of the stimulated 6 PY neurons projecting to all RE neurons (yellow asterisk). In both cases, RE membrane potential was depolarized for the duration of RE current injection or PY stimulation (purple asterisks), but this did not correspond to an increase in RE spiking (orange asterisks). Like the spontaneous KC (Figures 4 & 5), a drop in the TC and RE spindling occurred at the same time as the evoked KC (red asterisks) in both cases.

**Figure 8:**
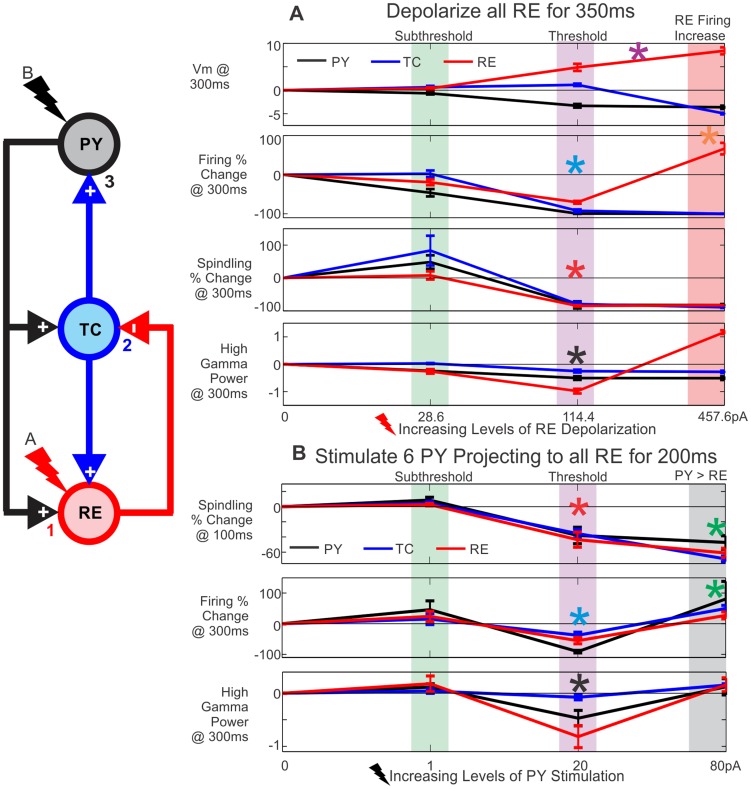
Parametric analysis of evoking KCs by directly versus indirectly depolarizing RE. The effect of increasing levels of RE depolarization or PY stimulation was inspected at individual time points. The schematic to the left outlines the connectivity and the order of effects on: 1) RE neurons, 2) TC neurons, and 3) PY neurons, based on RE depolarization (A) or PY stimulation (B). Five simulations with different random seeds were run for each value plotted, as well as a no stimulation baseline run. Baseline correction was performed for membrane potential (Vm) and high gamma power. Percent change was calculated for spindling power and firing. The average of five runs at a particular stimulation value and time point is plotted ± SEM. A, As the level of applied RE depolarization increases, so does the RE membrane potential (purple asterisk); however, RE firing does not increase until high levels of depolarization are reached (red box and orange asterisk). The green box highlights a level of RE depolarization that is subthreshold for evoking KCs. At 114.4pA (purple box), PY firing and high gamma power drop (blue and black asterisks, respectively), indicating a KC. Spindling also decreases in all three cell populations (red asterisk). The red box outlines a level of stimulation where RE firing increases, leading to the production of a KC. B, When stimulating 6 PY neurons projecting to all RE neurons, the spindling in all three populations drops 100 ms after stimulation is applied at a level of 20pA or higher (red asterisk). At 20pA (purple box), the firing in all cell populations (blue asterisk) and high gamma power in PY neurons (black asterisk) drop 300 ms after stimulation, indicating a KC. The green box highlights a subthreshold level of PY stimulation. At 80pA of PY stimulation (grey box), there is a drop in spindling but no KC, presumably because the direct cortical excitation is sufficient to counteract the removal of thalamic input (green asterisks).

In addition to occurring spontaneously, KCs may be evoked by sensory stimuli in humans. We tested whether KCs could be evoked in the model in two ways: (1) by directly depolarizing all RE neurons in the baseline model where each PY neuron projects to 5 RE neurons; or (2) by stimulating the subset of PY neurons which project to all RE neurons. Each allowed direct testing of a part of the PY-RE interaction observed in a spontaneous KC by intervening at different stages of the network. We first illustrate the effects of depolarization with a detailed example (Figure 7) and then provide summary data from parametric analysis (Figure 8).

As predicted by our hypothesized mechanism, a KC was reliably evoked by injection of a depolarizing current into all RE neurons in the baseline model. These events were identified as KCs by a drop in the PY membrane potential and decreased PY spiking (Figure 7A, blue asterisks and “KC”). The orange box marks the 350 ms duration of depolarization applied at 85.8pA. We had expected RE depolarization (Figure 7A, purple asterisks) to lead to increased firing in the RE neurons; surprisingly, this was not the case. RE firing actually decreased during most levels of RE depolarization tested (see Figure 7A, orange asterisk, for an example and Figure 8A for summary data). In seeking the mechanism of this counter-intuitive result, we noted that the RE depolarization systematically leads to RE and TC spindling disruption (see Figure 7A, red asterisks, for an example and Figure 8A for summary data). Note that we had observed a similarly decreased spindling in the spontaneous KCs. These processes, including the cortical KC, start at a short latency (<200 ms) after the onset of RE depolarization, and thus do not represent a post-depolarization rebound phenomenon.

We then tested the reconfigured model described above where 6 of the PY neurons project to all RE neurons at the baseline strength of 0.5 µS (Figure 3C). Depolarization of these widely projecting PY neurons provided a more direct test of our hypothesized mechanism. As predicted, directly depolarizing the 6 widely-projecting PY neurons triggers a KC (see Figure 7B for an example and Figure 8B for summary data). The black box in Figure 7B delineates the 200 ms duration of depolarization applied at 15pA to these 6 PY neurons. The KCs evoked in this manner are characterized by a drop in both the PY membrane potential and the PY spiking (Figure 7B, blue asterisks and “KC”). They thus closely resemble the electrophysiological characteristics observed in spontaneous KCs (Figures 4 & 5) and KCs evoked by direct RE depolarization (Figures 7A & 8A). As expected, the 6 PY neurons indicated by a red arrow that project to all RE neurons are depolarized before the KC (Figure 7B, yellow asterisk). As in the case of directly depolarizing all RE neurons, the RE neurons are also depolarized when stimulation is applied to the 6 PY neurons that project to all the RE neurons (see Figure 7B, purple asterisks for an example and Figure 8B for summary data). In this case as well, the RE depolarization is not accompanied by increased RE firing (Figure 7B, orange asterisk for example and Figure 8B for summary data), but in most cases by decreased RE firing. As was observed after direct RE depolarization, stimulation of the widely projecting PY neurons also disrupted TC and RE spindling (Figure 7B, red asterisks for an example and Figure 8B for summary data).

#### Parametric analysis supports a critical role of TC and RE spindle disruption in KC generation

The simulations described above support the hypothesis that a lack of drive from TC neurons to PY neurons can trigger both spontaneous and evoked KCs. However, contrary to our hypothesis, this decreased drive did not occur due to increased firing in RE neurons. Rather, the consistent finding was that decreased TC firing reflected disrupted spindling by the TC-RE circuit.

In order to systematically investigate the relationship between cortico-thalamic input to RE, spindling in RE and TC, TC firing, and KCs, we examined different baseline corrected properties of the network response over a range of RE depolarization (Figure 8A) and PY stimulation (Figure 8B) values. Each point is the mean of five baseline corrected runs ± standard error of the mean (SEM) (see Methods, baseline corrected values). For each network property, the PY (black), TC (blue), and RE (red) values are plotted together. The basic network diagram to the left of Figure 8 provides color coding for the cell types and illustrates the effects of RE depolarization (red bolt, A) or PY stimulation (black bolt, B) on 1) RE neurons, 2) TC neurons, and 3) PY neurons, ultimately leading to a KC.

In the case of directly depolarizing all RE neurons, there is a depolarization threshold–both in terms of duration (not shown) and amplitude –for evoking KCs (Figure 8A). The points plotted occur 300 ms into the 350 ms of RE depolarization. The RE Vm directly reflects the increasing amount of applied depolarization (Figure 8A, purple asterisk). RE firing initially decreases with increasing RE depolarization (Figure 8A, red line, blue asterisk), and only increases when a high level of depolarization is applied (Figure 8A, orange asterisk). The purple box highlights a threshold level of depolarization to produce a KC characterized by a drop in PY firing (blue asterisk) and HGP (black asterisk). A green box highlights a subthreshold depolarization, while the red box outlines a KC produced when RE depolarization is strong enough to increase RE firing. Therefore, there are two ways in which RE could affect TC firing–via spindling suppression from increasing depolarization of thalamic neurons and via direct TC inhibition from a depolarization high enough to increase RE spiking, as originally hypothesized.

Figure 8B examines the stimulation of 6PY neurons projecting to all RE neurons at different levels of stimulation. In the top panel of Figure 8B, the points plotted occur 100 ms into the 200 ms of PY stimulation. At this time point, stimulation at 20pA or 80pA leads to a drop in both TC and RE spindling (Figure 8B, red asterisk). The bottom two panels of Figure 8B correspond to time points that occur 100 ms after PY stimulation has ended (300 ms from the beginning). At 20pA, there is a KC characterized by a drop in PY firing (Figure 8B, blue asterisk) and PY HGP (Figure 8B, black asterisk). The green box highlights a subthreshold level of PY stimulation. In this configuration, drops in TC and RE spindling can be indicative of a KC; however, there is not an absolute threshold as was the case with depolarizing all the RE neurons. For example, at 80pA there was a drop in TC and RE spindling at 100 ms that did not result in a KC at 300 ms (Figure 8B, green asterisks). This may be explained by a depolarizing effect of direct PY stimulation and the rebound in TC neurons at the stimulus offset. Thus, while all KCs that we observed had disrupted RE and TC spindling, the converse was not true: a RE and TC spindling decrease was not always predictive of a KC. However, this should be considered a strong exception to the otherwise universal association of KCs with decreased spindling, because it was only observed with high levels of PY stimulation, which had additional effects of directly activating TC neurons as well as other PY neurons. In summary, parametric analysis of this model supports a strong association between disruption of spindling and KCs, mediated by removal of TC-PY activation.

#### Inactivation of RE I_T_ underlies RE and TC spindling disruption that leads to a KC

**Figure 9:**
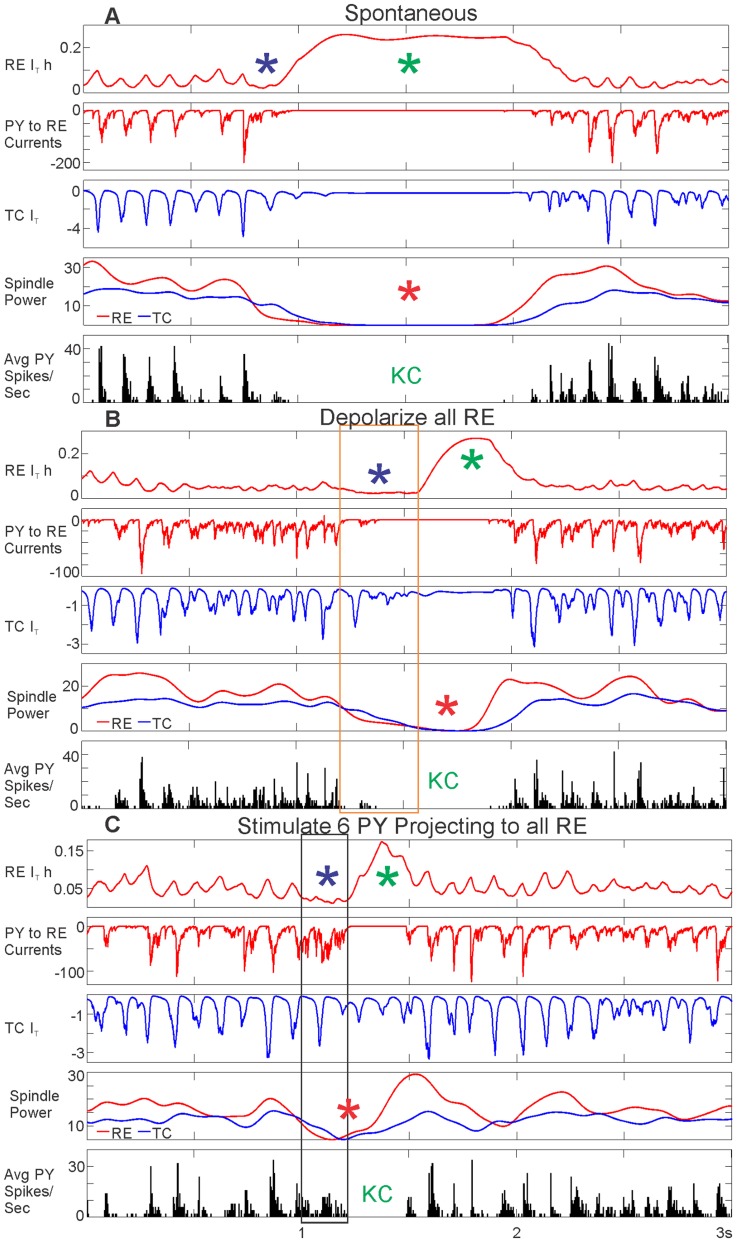
Currents and conductances underlying KCs. A, A spontaneous KC (same KC as in Figure 4), B, a KC evoked by depolarizing all RE neurons (same KC as in Figure 7A), or C, a KC evoked by stimulating 6 PY neurons projecting to all RE neurons (same KC as in Figure 7B), all show a drop in PY spiking (“KC”), and in RE and TC spindling (red asterisks). The orange box in B and the black box in C indicate the length of the applied RE depolarization or PY stimulation, respectively. In all three cases, these characteristics of the KC were preceded by RE I_T_ inactivation dropping towards zero (RE I_T_ h, purple asterisks), indicating greater inactivation. A decrease in PY to RE currents and a decrease in TC I_T_ accompanied this RE I_T_ inactivation drop. During the KC, an increase in RE I_T_ h indicates greater deinactivation (green asterisks), and rebound spindling.

We next sought to understand the underlying mechanism of the TC and RE spindling disruptions critical to generating both spontaneous and evoked KCs. We hypothesized that because the low-threshold Ca^2+^ current, I_T_, in both RE and TC neurons is crucial for spindle generation [41]–[43], and because it is easily inactivated by RE depolarization, that it may mediate the observed effects. Specifically, we hypothesized that depolarization of RE would inactivate I_T_ and this would prevent spindling from occurring. Furthermore, since I_T_ is responsible for burst firing during spindles, this would decrease the overall firing of RE neurons during depolarization. Figure 9 compares the spontaneous KC from Figure 4 and the evoked KCs from both depolarizing all RE neurons and stimulating a subset of 6 PY neurons projecting to all RE neurons from Figure 7. We examine RE I_T_ inactivation (by monitoring the inactivation variable, h, of the I_T_ current, RE I_T_ h), PY to RE currents, and TC I_T_, in comparison to spindling power and PY spiking in all three cases.

I_T_ is fully inactivated when RE I_T_ h drops to zero. For all three KCs, RE I_T_ h first decreases towards zero (Figure 9, purple asterisks), which is followed by a drop in RE and TC spindling (Figure 9, red asterisks) and PY spiking (Figure 9, “KC”). For the evoked KCs, this drop in RE I_T_ h occurred during the period of RE depolarization (Figure 9, orange box) or PY stimulation (Figure 9, black box). During the KC, RE I_T_ h increases as it becomes deinactivated (Figure 9, green asterisks), and ultimately spindling resumes, as does firing, in all populations.

**Figure 10:**
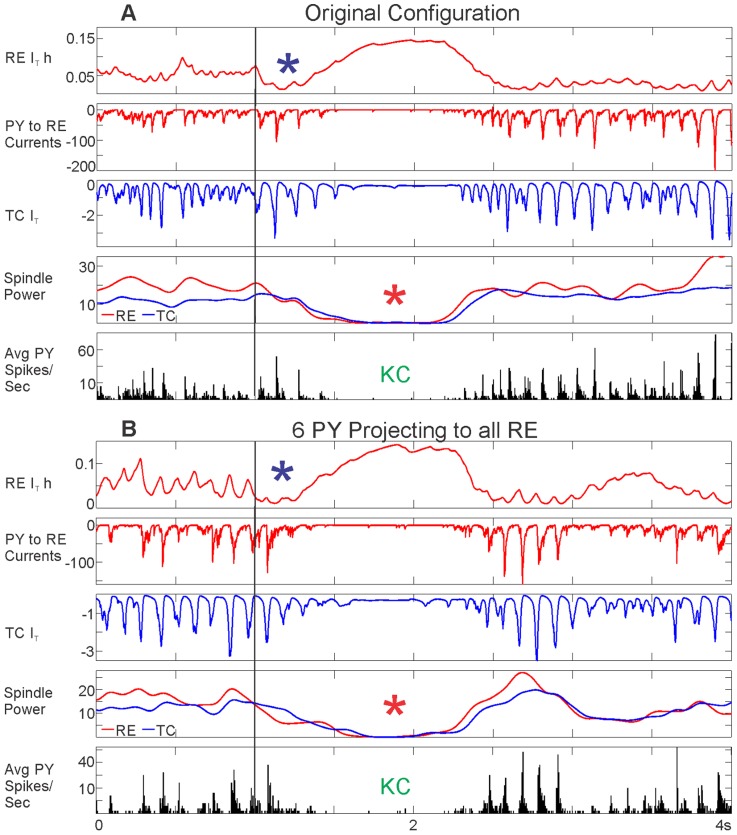
Inducing KCs by abruptly increasing RE I_T_ inactivation (RE I_T_ h). A, In the original configuration and, B, in the 6 PY neurons projecting to all RE neurons configuration, RE I_T_ h is scaled to 40% of its original value (purple asterisks) at 1 s (vertical black line). In other words, the proportion of deinactivated I_T_ channels is abruptly decreased. In both cases, this drop in RE I_T_ h leads to a decrease in PY to RE currents and TC I_T_, as well as a drop in RE and TC spindling (red asterisks), which ultimately leads to a KC, as indicated by decreased PY spiking (“KC”).

**Figure 11:**
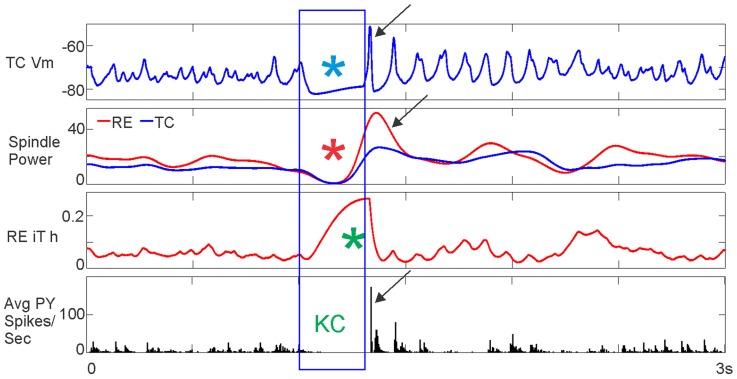
Hyperpolarizing TC neurons produces a KC by decreasing the thalamocortical drive to cortex. All TC neurons were hyperpolarized at 116pA for 300 ms. The length of TC hyperpolarization is outlined by the blue box. For the duration of TC hyperpolarization (blue asterisk), RE and TC spindling dropped (red asterisk), RE I_T_ became deinactivated (green asterisk), and PY spiking dropped to zero (“KC”), indicating a KC. This was followed by a rebound upstate, as marked by TC depolarization, increased RE and TC spindling above baseline levels, and high PY spiking (black arrows).

In order to directly test the hypothesis that RE I_T_ inactivation produces a KC by disrupting RE and TC spindling, we attempted to induce a KC by abruptly increasing the amount of RE I_T_ inactivation. RE I_T_ h was scaled to 40% of its original value to test if it would result in a drop in RE and TC spindling and a KC, in both model configurations used to evoke KCs (Figure 10). The black vertical line indicates the time point at which RE I_T_ h was scaled to 40% of its value in both the original configuration (Figure 10*A*) and the configuration where 6 PY neurons project to all RE neurons (Figure 11*B*). The sudden increase in RE I_T_ h (visualized as a drop towards zero) is seen in both cases (Figure 10, purple asterisks). This leads to a drop in RE and TC spindling (Figure 10, red asterisks) and PY spiking (Figure 10, “KC”), indicating a KC. The scaling of RE I_T_ h also leads to accompanying drops in PY to RE currents and TC I_T_.

In the case of both spontaneous and evoked KCs, RE I_T_ inactivation causes a disruption of TC and RE spindling that decreases the drive to PY from TC, leading to a cortical KC characterized by a drop in membrane potential, spiking, and HGP in PY neurons. In the case of evoked KCs, RE I_T_ inactivation is triggered by RE depolarization, either directly or from a stimulated group of widely-projecting PY neurons.

#### KCs are produced by a lack of drive from TC neurons to PY neurons

Our findings above provide evidence that decreased TC and RE spindling driven by I_T_ inactivation leads to a KC due to disruption of the thalamocortical drive to the cortex. In order to directly test the hypothesis that a lack of drive from TC neurons to PY neurons can trigger a KC, we hyperpolarized all TC neurons. In Figure 11, 116pA of hyperpolarization for 300 ms is applied to all TC neurons. The top panel shows a clear hyperpolarization in TC (Figure 11, blue asterisk). This produces a disruption of RE and TC spindling (Figure 11, red asterisks) and a complete lack of cortical firing (Figure 11, “KC”), as was seen in all previous cases of KCs. During this period, we also see RE I_T_ deinactivation (Figure 11, green asterisk), as pictured in Figures 9 and 10. As opposed to previously examined KCs, RE I_T_ does not become more inactivated preceding the KC, because we are directly hyperpolarizing the TC neurons. Furthermore, the lack of PY firing and the spindle disruption only lasts as long as the application of TC hyperpolarization. Once the hyperpolarization is removed, the KC is followed by a rebound increase above baseline of TC Vm, RE and TC spindling, and PY spiking (Figure 11, black arrows). TC hyperpolarization triggering a cortical downstate for the duration of the hyperpolarization is consistent with our previous findings, except in those cases, depolarization of RE neurons hyperpolarized the TC neurons. Strongly depolarizing RE neurons that then inhibit TC neurons is one way in which this hyperpolarization could occur (Figure 8). More commonly, suppression of firing in TC neurons was accomplished by weakly depolarizing RE neurons, which depresses spindling in RE and TC, leading to a cortical KC (Figures 7 & 8). Hyperpolarization of TC was tested over a range of levels (not shown). As the level of hyperpolarization increased (became more negative), a downstate would occur during the time of hyperpolarization, followed by a rebound upstate as pictured in Figure 11, but the system could not recover and was followed by a long-lasting downstate. These findings demonstrate that hyperpolarization of TC neurons is sufficient to produce a KC.

#### Depolarizing or hyperpolarizing other populations in the model does not produce KCs

Using the original model configuration, we also tested whether depolarizing or hyperpolarizing the other neuronal types could trigger KCs. We found that depolarizing TC or PY neurons only lead to a downstate after an upstate. Depolarizing inhibitory interneurons (IN) could trigger a downstate, but the downstate only lasted as long as the depolarization was applied to the IN. KC generation using this mechanism would require a widespread projection to the IN.

### Simultaneous SEEG Recordings from Thalamus and Cortex during Sleep

#### Thalamic spindling power drops before and during frontal KCs

**Figure 12:**
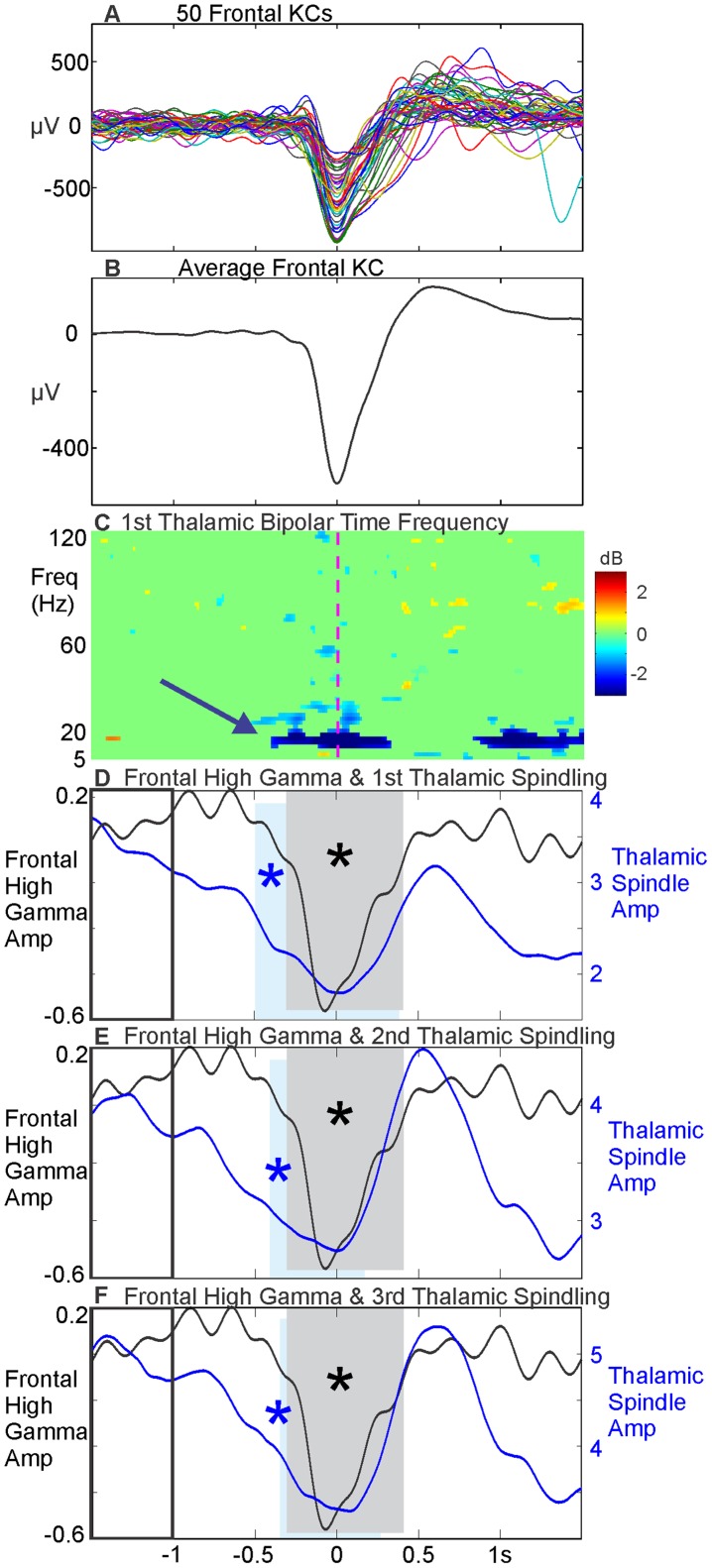
Disruption of spindling in the human thalamus precedes the cortical KC. KCs chosen on a single prefrontal bipolar SEEG channel located in Brodmann's area 10 are displayed as single trials (A, 50 randomly selected individual KCs) and, B, the average of all 229 KCs, band pass filtered to 0.1 to 5 Hz. C, Time frequency analysis (5–120 Hz) in the 1^st^ (most medial) thalamic bipolar SEEG channel using the times of the frontal KCs in B, thresholded at p<0.01 (uncorrected) compared to the −1.5 to −0.5 second baseline. The blue arrow shows that there appears to be a drop in spindle power. D, The average absolute value of the Hilbert transform applied to the frontal KCs in B, band pass filtered for high gamma (60–120 Hz), is plotted in black. The grey box indicates the time period where high gamma drops significantly compared to baseline, outlined with a black box (−1.5 to −1 seconds, p<0.01, FDR corrected). The blue line indicates the average absolute value of the Hilbert transform applied to the 1^st^ thalamic bipolar SEEG channel band pass filtered for spindling (12–16 Hz), using the times of the frontal KCs. The blue box indicates the time period where spindling drops significantly compared to baseline (p<0.01, FDR corrected). The drop in thalamic spindling (blue box) occurs prior to the drop in cortical high gamma (grey box). E& F, The same analysis as outlined in D is applied to the 2^nd^ thalamic bipolar channel (E) and the 3^rd^ thalamic bipolar channel (F). The cortical data are the same in all three subplots; the thalamic spindling amplitude scales are individualized for each thalamic bipolar contact in D–F. For all three thalamic bipolar channels, thalamic spindling drops significantly (blue box) prior to the significant cortical high gamma drop (grey box).

As we cannot intracellularly measure the human cortex or thalamus to directly test whether RE I_T_ inactivation causes spindle disruption leading to KCs, as suggested by the model, we sought instead to empirically test the model prediction that thalamic spindle disruption precedes the KC. We tested this hypothesis using rare simultaneous bipolar SEEG recordings from a patient with temporal lobe epilepsy, who had electrodes implanted in the prefrontal cortex and the thalamus (Patient 5). During N2 and N3, we selected 229 KCs on a prefrontal bipolar SEEG channel estimated to lie in Brodmann's area 10. The KCs chosen on this channel exhibited a classic KC morphology, without a preceding upstate, on an individual trial and averaged basis (Figure 12A & B). Using the times of the frontal KCs, a time frequency analysis (5–120 Hz) was performed on the 1^st^ thalamic channel (Figure 12C) and there appeared to be a drop in thalamic spindling power preceding the prefrontal KC (blue arrow in Figure 12C). In order to test whether this drop was statistically significant across trials, we first accurately identified the onset of the KC by measuring the drop in local high gamma activity. Amplitude of the prefrontal bipolar SEEG from 60–120 Hz was measured during each trial using the Hilbert transform. This amplitude was compared at each point to a baseline period (−1.5 to −1 s, outlined by a black box in Figure 12D–F, before the KC peak at time zero), and the period of significant (p<0.01, FDR corrected) drop in high gamma was identified. The period of cortical high gamma drop identified in this way is highlighted by a grey box in Figure 12D, E & F. We then identified the onset of the thalamic spindling drop in the same manner for the 1^st^ (Figure 12D), 2^nd^ (Figure 12E), and 3^rd^ (Figure 12F) thalamic bipolar channels (with the 1^st^ thalamic channel being the most medial). The amplitude of each thalamic bipolar channel was measured from 12–16 Hz continuously on each trial using the Hilbert transform and compared to a baseline period (−1.5 to −1 s) in order to identify periods of significant (p<0.01, FDR corrected) drop in thalamic spindling. These significant periods of thalamic spindling drop are outlined for each individual thalamic bipolar channel by a blue box in Figure 12D–F. The scale for the thalamic spindling amplitude is individualized for each bipolar contact to better visualize the significant drop in each. In all three thalamic bipolar channels, the drop in thalamic spindling (blue box) occurs prior to the drop in cortical high gamma (grey box). We also tested all grey matter bipolar channels along the electrode targeting the thalamus (but lateral to the thalamus) and did not find any statistically significant decreases in thalamic spindling prior to the cortical high gamma drop. While this result is consistent with the computational model, further studies are needed to determine its reliability in different patients and thalamocortical regions, given their high anatomical and physiological variability.

## Discussion

We describe a neural model for KCs during N2 sleep with detailed conductances and synaptic connections for the major cell types in the cortex and the thalamus. KCs in this model are cortical downstates, without a preceding upstate, that occur quasi-synchronously across the cortex, either spontaneously or evoked by a variety of external stimulations. These cardinal features of human KCs have been empirically demonstrated in this and previous studies [5]–[7]. Analysis of simulations with this model yielded an unexpected result: KCs were systematically preceded by an induced or spontaneous decrease in thalamic spindling. The decrease in spindling was caused by a depolarization-induced inactivation of I_T_. Decreased thalamic spindling caused an abrupt decrease of thalamocortical excitatory drive, leading to the synchronous cortical downstate. We then tested the main prediction of this model in a simultaneous recording from the human thalamus and cortex in a single patient; we found that, indeed, decreased thalamic spindling preceded cortical KC downstates.

The first cardinal feature of KCs reproduced in the current model is that they occur intermingled with sleep spindles, as is characteristic of human N2 [6], [7]. Thus, our model includes the thalamic and cortical circuits and currents crucial for generating spindles [41]–[43], together with the intracortical circuits which are crucial for SO generation [2], [14], [16], [22]. Although cortico-cortical connections are sufficient for generation of rhythmic upstates and downstates, thalamocortical interactions have been increasingly implicated in the synchronization and control of SOs [29], and thus may play a similar role for the KC. Indeed, our model suggests that thalamocortical and corticothalamic connections may both play key roles in triggering KCs. As expected from the strong projection of TC neurons to the cortex, directly hyperpolarizing TC neurons abruptly removes excitation from cortical neurons, causing a KC. Similarly, as expected from their known synaptic connections, strong RE activation directly inhibits TC neurons, and the model found that this results in a KC, due to abrupt removal of TC excitatory drive from the cortex. However, at low levels of depolarizing input to RE neurons, these neurons may actually decrease their firing rate, yet a KC is still observed. Analysis of the model found that this apparently anomalous result occurs because weak depolarization of RE inactivates I_T_, which disrupts thalamic spindling. Since burst firing during spindling is the main activity of TC projection neurons in N2, the disruption of spindling causes a profound suppression of firing, thus triggering KCs.

A second cardinal feature of KCs produced by our model is the generation of KCs without preceding upstates. In previous models, downstates arose as the direct product of a preceding upstate, resulting, for example, from synaptic exhaustion or inward potassium currents due to high activity [2], [3], [13]–[15]. Such mechanisms may be important for downstates during the SO of N3. However, they cannot explain the KC because it is not preceded by an upstate. Conversely, the mechanism modeled here could contribute not only to KC generation, but also to synchronization during the SO [24]. We identified cortical KCs in PY neurons by their hyperpolarized membrane potential, decreased high gamma power, and decreased firing rate, with the opposite changes identifying upstates. By these criteria, our model generates KCs without preceding upstates, as is observed in the human cortex during KCs [5], and at a rate consistent with empirical observations in humans [38]–[40].

The third cardinal feature of KCs, demonstrated by our empirical recordings and reproduced by our model, is the quasi-synchrony of KCs across the neocortex. When we began this study, it was unclear whether KCs should be modeled as quasi-synchronous downstates or as travelling waves. On the one hand, qualitative observation of scalp EEG suggested that KCs may be synchronous across the scalp [6]. On the other hand, the prevailing view in the field, based on scalp EEG, is that SOs are travelling waves [17] , and thus KCs, being composed of the downstate segment of SOs, are also. However, scalp EEG patterns do not uniquely determine their underlying cortical generators, and they are particularly ambiguous when sources are highly distributed, due to smearing across the skull and source cancellation [44]. Furthermore, since the activity of multiple cortical components are superimposed in each scalp EEG sensor, apparent latency differences between different sensors may actually arise from different proportions of underlying components with fixed latencies. Thus, it is important to refer to direct cortical recordings to resolve this issue. Using dual intracellular recordings from cells separated by 4 to 12 mm, Volgushev *et al.* [45] found that the onset of the downstate in anesthetized cats propagated on average at ∼7 m/s. In contrast, Sanchez-Vives and McCormick, 2000, found a propagation speed of 0.011 m/s in cortical recordings from ferret slices [12]. While Volgushev *et al.* considered that the onsets were essentially synchronous, their conduction velocity was close to the range reported by Massimini *et al.* as evidence that the SO are travelling waves: between 1.2 and 7 m/s on the scalp, with an approximate 120 ms delay from frontal to occipital EEG recording sites [17]. Given the broad range in conduction velocities and terminology in these studies of the SO, and their questionable applicability to human KCs in natural sleep, we also considered the literature on travelling waves in the occipital cortex of animals during visual processing, which have conduction speeds in the range of 0.1–0.4 m/s [34], or up to 0.6 m/s [35]. We found that the latencies of KC peaks across the frontal and occipital scalp in EEG, or between frontal and parietal cortex in ECOG and SEEG, indicate an average delay that would correspond to propagation speeds at least 10 times faster compared to scalp EEG estimates [17], about 2000 times faster compared to in vitro estimates [12], about 4000 times faster than modeling estimates [14], and about 30–200 times faster than those observed in cortical travelling waves [34]. Based on these empirical observations of the short delays between averaged KCs recorded at distant sites, we attempted to model KCs as quasi-synchronous rather than cortical travelling waves. We also provide examples of recorded single KCs which are quasi-synchronous, and thus our results with average KCs do not arise from combining KCs with relatively large latency differences. However, such KCs do exist, and it remains a challenge for future research to determine if the apparent variability in the sequence of KC onset across sites reflects measurement error due to superimposed noise, propagation of slow activity under certain circumstances, or a shifting focus of readiness to produce KCs as has been suggested for the SO [17], [20], [45].

Consistent with our results, Wennberg, 2010 [46] reported that KCs recorded with SEEG during normal sleep in humans appeared to be synchronous, although he did not quantify their relative latencies. In contrast to our findings, he reported dramatic frontal to posterior amplitude decrements, with phase reversal in the temporal lobes, which he interpreted as indicating that ventral temporal and occipital areas do not generate KCs. Wennberg, 2010 recorded SEEG relative to an average reference. It can be difficult to determine with certainty whether an SEEG contact is in the subdural space or within the cortex, and this can have very large effects on SEEG amplitude and polarity. Similarly, an average reference constructed during a very widely generated graphoelement would itself generally be large, unless it is constructed from a complete sampling of the surface bounding the generating structures, which is not possible in this case. In contrast to Wennberg, 2010, we either recorded ECOG, which has a consistent location relative to the grey matter imposed by physical constraints, or from bipolar transcortical SEEG, which must be locally generated given the amplitudes that we observed. Nir *et al.*, who recorded SEEG relative to a largely inactive reference (linked earlobes), found KCs in ventromedial temporal limbic structures associated with decreased unit firing, supporting local generation [20]. Together with our observations, these data suggest that ventral temporal structures also generate KCs. Nir *et al.* also found that slow waves in the medial temporal lobe limbic cortex systematically followed those in medial frontal cortex by about 180 ms [20]. This difference from our results could reflect the fact that their observations were mainly of SOs, or that they were between cortical and limbic areas rather than between neocortical locations. Nonetheless, significant variability is observed in the local amplitude of KCs across sites and trials, which remain to be systematically explored.

A fourth remarkable cardinal feature of KCs reproduced by the model is that they can be either spontaneous or evoked by a variety of sensory stimuli. Our previous findings showed that both are cortical downstates [5], but there remains controversy regarding whether they represent the same neurophysiological phenomenon [47], [48]. In particular, the original observation made by the Loomis laboratory that different modality stimuli can evoke KCs has led to the investigation of modality-specific evoked KC responses. Modality-specific modulation of the topography or source localization of the P200 component of the KC for auditory, visual, and somatosensory stimuli has been reported [48], [49]; however, it is difficult in these studies to disentangle the effects of superimposed modality-specific (sensory) responses onto non-modality-specific P200 activity (i.e., the early phase of the KC). For auditory evoked KCs, our current results in Figure 1B–F show that spontaneous and evoked KCs have the same topography and highly correlated amplitudes across the cortex, at a resolution unachievable with scalp recordings.

Some studies have shown that the efficacy of different stimuli in evoking KCs varies with their complex cognitive characteristics, but this remains controversial (reviewed in [6], [7]). If such complex control over evoked KCs does exist, then it would suggest that there is a cortical control mechanism over the triggering of KCs; for our schema, that would imply a cortico-RE projection, such as has been found from focal prefrontal areas (Brodmann 9, 13, and 46) to widely distributed areas of the RE in rhesus monkeys [31]. We tested the hypothesis that excitation of these focal prefrontal areas would broadly excite the RE neurons, thus resulting in a synchronous isolated KC. We found that the addition of such connections from a restricted cortical area to the RE was required for spontaneous KCs to occur in our model, and further, that spontaneous KC frequency was proportional to the intensity of such connections. Recently, a parallel pathway from the amygdala to widespread RE sites has been reported [50]. The amygdala and posterior orbital cortex (including area 13) are thought to interact in assigning emotional valence to stimuli [50]. It is an intriguing possibility that they also interact during sleep in evaluating whether stimuli should be permitted to arouse the subject.

We noted above that an unexpected finding of our simulations was that all KCs showed disruption of TC and RE spindling, which correlated with RE I_T_ becoming more inactivated. Evoked and spontaneous KCs were triggered when the excitatory drive to the cortex from the thalamus was abruptly decreased, consequent to sufficient excitation of the RE neurons to inactivate RE I_T_. Furthermore, making RE I_T_ more prone to inactivation led to an immediate disruption of thalamic spindling and a KC. The role of I_T_ in spindle generation is well known [51] and recent work has shown that thalamic network dynamics are highly sensitive to the inactivation status of I_T_ [52]. Spindling is also more sensitive to knocking out the T-type Ca^2+^ channel subtype abundantly expressed in RE neurons (Ca*_v_*3.3), than to knocking out the subtype exclusively expressed in TC neurons (Ca*_v_*3.1) [53], [54].

The interaction between spindles and KCs has long been of interest [6], [55]–[58]. Our model predicts that thalamic spindling would decrease before and during KCs, but the implications of the model for scalp EEG, which reflects cortical spindles, are uncertain. As our model predicts for thalamic spindles, scalp spindles do not occur during KCs [59], [60]. However, the presence and termination of scalp spindles prior to KCs is not strongly regular. For example, it has been demonstrated in scalp EEG that only ∼30% of KCs are preceded by spindles while ∼70% of KCs are followed by spindles [60]. Furthermore, KCs elicited by stimuli delivered during, versus away from scalp spindles, did not show the differences in either rate or amplitude [55] that one might expect from the model, although this was not simulated, so the implications for the model are not known. Although MEG spindles are also generated in the cortex, it may be useful to consider them in order to infer the properties of thalamic spindles. Whereas spindles in scalp EEG are well-delineated discrete events that occur only several times per minute [61], MEG spindles commonly occur without EEG concomitants, in a quasi-continuous fashion during N2 [18], similar to SEEG [26]. It appears that spindles in focal cortical generators may be detectable in MEG, but not in EEG until multiple focal generators become synchronized and involve more diffuse generators, thus recruiting a sufficient cortical domain to become visible in scalp EEG [18], [26]. Since the MEG spindles imply thalamic spindles, EEG would seem to give an incomplete sampling of when thalamic spindling occurs, and thus would not provide a strong test of the model. The necessary simultaneous SEEG recordings from thalamus and cortex during natural sleep are rare, but we were able to perform such recordings in one patient. As predicted, a simultaneous SEEG recording of KCs from prefrontal cortex and spindling from the thalamus found that spindle power in all bipolar contacts in the thalamus decrease before the KC.

The interaction of spindles and KCs may be important for memory consolidation. The organization of memory replay during spindles and KCs is unknown, but the temporal coordination of the SO upstate, fast spindles, and hippocampal sharp waves has been implicated in memory consolidation [8], [9]. One can speculate that a recursive rhythmic thalamocortical interaction evolves during each spindle discharge to consolidate a particular memory. Over the course of the spindle, intracellular Ca^2+^ accumulates, facilitating plasticity, but eventually causing inactivation and termination of the spindle [23], [41], [62]. Our model indicates that this would also evoke a KC in some cases. The KC downstate would shut down most thalamocortical neural activity, followed by rebound spindling. The KC may therefore provide closure to the consolidation of one memory before the following spindle launches consolidation of another memory. Indeed the increased coordination of upstates, downstates, and spindles has a powerful effect on memory consolidation [63].

Although our model was arguably realistic in terms of active channels, synaptic currents, cellular dynamics, cell types, and the basic connections between them, it is obviously limited in the number of modeled neurons. A distinction was not made between slow spindles seen maximally in frontal areas and fast spindles seen maximally in parietal areas in scalp EEG; these separate spindle categorizations would require a much larger model. In a related fashion, our model also did not distinguish between matrix and core thalamocortical systems [64]. Matrix projections are more widespread than core, and modeling and empirical evidence suggest that they are important for synchronizing EEG sleep spindles [18], [19], [23], [26]. Additional synchronization would be expected in our model if the posited cortico-thalamic projections preferentially targeted TC matrix neurons. We also failed to model the diffuse projections from brainstem monoaminergic structures such as the locus coeruleus to the cortex [65], although their role in KC generation is unlikely because they fire at low rates during SWS and their activation lasts much longer than the KC. We also did not model direct inhibition of PY via a long range GABAergic inhibition from the basal forebrain [66], [67] or a diffuse cholinergic projection to the IN, whose excitation could conceivably lead to widespread indirect inhibition of PY neurons [67], [68]. When we modeled the effects of widespread excitation of the IN, we found, as expected, that PY were inhibited as in the KC. However, the PY inhibition did not significantly outlast the IN excitation, which is inconsistent with empirical observations that both cell types are silent during the downstate in humans [4], [5], [69] and animals [16]. Nonetheless, this possibility deserves further exploration with GABA_B_-mediated PY inhibition, which would greatly outlast the IN excitation [70]. This mechanism would make the empirical prediction that at least some of the IN are activated immediately prior to the KC and may be responsible for the synchrony leading to the KC [16].

In summary, we describe here the first neuronal model that reproduces the four cardinal characteristics of the KC: (1) KCs occur together with spindles in N2; (2) KCs are isolated downstates without a preceding upstate; (3) KCs can appear quasi-synchronously across multiple lobes in both hemispheres; and (4) KCs can occur both spontaneously and be evoked by a variety of sensory stimuli, with nearly identical mechanisms and distribution. We present novel empirical evidence to constrain the model from recordings made directly from the human cortex. The model demonstrates a possible mechanism whereby widespread quasi-synchronous cortical downstates (KCs) may be triggered by the disruption of thalamic spindling. The prediction of disrupted thalamic spindling prior to KCs was tested and observed in rare simultaneous cortical and thalamic human recordings; however, the further precise mechanism predicted by the model, involving inactivation of I_T_ by a depolarizing input to RE neurons from a small cortical area, can only be fully tested in animal models.

## Materials and Methods

### Ethics Statement

The institutional review boards of The Children's Hospital, Boston, and Partners Healthcare Inc., in addition to the ethics committee of Comité Consultatifs de Protection des Personnes se Prêtant à des Recherches Biomédicales Lyon-Centre Léon Bérard, approved the parts of this study conducted at each respective site. Written consent was obtained directly from all patients.

### Empirical Measures

**Cortical intracranial recordings** were obtained in patients (three women, one man) suffering from pharmaco-resistant epilepsy who were candidates for surgical resection of their seizure focus, preceded by intracranial EEG (iEEG) for localization of that focus, at Massachusetts General Hospital, Brigham and Women's Hospital, and Children's Hospital. The electroencephalogram (EEG) and electrooculogram (EOG) were recorded simultaneously with iEEG, which could be either the stereoencephalogram (SEEG) or the electrocorticogram (ECOG). At all programs, electrodes are localized with respect to anatomical structures using CT with the electrodes in place, and intraoperative photographs [33]. Depth probes (SEEG) either had 6 contacts with 8 mm center-to-center spacing or 8 contacts with 5 mm center-to-center spacing. Each contact was 2.4 mm long with a diameter of 1.28 mm. The probes usually passed approximately perpendicular to the midsagittal plane. Subdural strip or grid electrodes (ECOG) usually included 8 contacts at 1 cm center-to-center, in 1 to 8 rows. 128–256 macro-contacts are recorded in each patient using cable telemetry systems and dedicated amplifiers. Fully informed consent was obtained prior to surgery under the auspices of local institutional review boards and in accordance with the Helsinki accords. The signals were sampled at either 500 or 256 Hz and then band-pass filtered from 0.1–120 Hz. Spontaneous KCs were recorded during natural NREM sleep, and KCs were also evoked during N2 with simple auditory tones that were presented randomly at 30–40 second intervals. Intensity was increased until more than half of the tones elicited a KC without arousal (see [5]).

**A thalamocortical intracranial recording** was obtained in one female patient suffering from pharmaco-resistant epilepsy at Neurological Hospital, Lyon, France. To delineate the extent of the cortical epileptogenic area and to plan a tailored surgical treatment, 12 SEEG recording electrodes were implanted according to the stereotactic technique of Talairach and Bancaud. Each electrode had ten to fifteen 2 mm contacts, with an inter-contact interval of 1.5 mm and a diameter of 0.8 mm. The medial pulvinar nucleus was a target of the thalamic implantation because its reciprocal connections with temporal cortical areas may be an important relay in the building of epileptic discharges. Furthermore, if the epileptic zone cannot be localized, this electrode placement allows a determination of whether stimulating the thalamus may decrease the frequency of seizures, with a view toward the eventual placement of a chronically implanted stimulator. Intracortical exploration of temporal neocortical areas and of the medial pulvinar nucleus was possible using a single multicontact electrode, so that thalamic exploration did not increase the risk of the procedure by requiring an additional electrode track. All patients were fully informed of the aim of this investigation and gave their written consent for the implantation and recording procedure, which was approved by the local ethics committee. The data were sampled at 256 Hz and a 0.33–128 Hz band pass filter was applied. Spontaneous KCs were recorded during NREM sleep.

**EEG recording** was obtained in one 23 year-old female subject during a full night recording. The subject was free of any neurological disorders, was not taking medication, and did not have caffeine or alcohol on the recording day. Sixty-four channels placed in a 10–10 montage were referenced to an average mastoid and sampled at 600 Hz.

### Patient Characteristics

One patient with ECOG and four patients with SEEG were analyzed (Table 1). Activity identified as epileptic or abnormal, including epileptiform KCs [71] and epileptiform spikes in conjunction with KCs [72], were excluded based on visual inspection.

**Table 1:** Patient demographics and clinical characteristics.

Patient	Gender	Age	Handedness	Electrode Type	Clinical Diagnosis	Pathological Diagnosis	Focus	IQ (FSIQ)	Intelligence
1	F	17	R	ECOG (Grids & Strips)	CPS; temporal lobe dysplasia	Temporal dysplastic lesion	Right temporal pole	75	
2	F	45	R	SEEG (Cortical)	CPS; Multifocal	Multifocal temporal parietal occipital	Temporal		Average
3	M	45	L	SEEG (Cortical)	CPS; bitemporal	No pathology obtained	Left and right mesial temporal lobes	83	
4	F	65	R	SEEG (Cortical)	CPS; Temporal lobe epilepsy with two foci: left mesial temporal structures & right subfrontal region	No pathology obtained	Right subfrontal & anterior temporal	101	
5	F	37	R	SEEG (Thalamic & Cortical)	Temporal lobe epilepsy	No pathology obtained	Hippocampus		Average

**Sleep staging and KC identification for cortical intracranial recordings** followed standard criteria and were verified by one qualified rater (SC). Sleep staging relies on iEEG, surface EEG (at least one scalp electrode referenced to the mastoid), and submental EMG, when available. N2 is identified by prominent KCs and spindles with only occasional delta activity in the absence of rapid eye movements [73]. N3 is classified with the onset of deeper sleep and the gradual diminishment of spindles and increase in delta frequency waveforms. KCs were selected based on multiphasic morphology during N2 or N3, occurring spontaneously or evoked by a sensory stimuli, with a significant surface negative and then surface positive potential occurring ∼500 and 900 ms from the beginning of the waveform [6]. Chosen KCs were isolated (i.e. not part of a preceding oscillation) and were not categorized based on preceding or following spindling activity.

**Sleep staging for the thalamocortical intracranial recording** was performed based on cortical activity across intracranial contacts and a scalp electrode by one qualified rater (HB).

**Bipolar SEEG analysis** was performed by examining voltage differences across bipolar depth electrode contacts that spanned the local cortical gray matter. Typically, one contact of the bipolar pair lay above the cortical gray matter, in the CSF, and the other just below it in the white matter. As KCs have such extensive generators, distant sources, although individually weaker than local sources, are so numerous that in sum they could contribute substantially to the recorded iEEG signal [32]. When many distributed generators are active, even extracranial locations serving as a reference lead may record high voltage responses. Our bipolar method obtains unambiguously focal cortical recordings free of the volume conduction and reference lead issues that may confound interpretation of iEEG. The surface negative component is usually dominant and corresponds to a downstate [5].

**KC detection for cortical intracranial recordings** was performed on the midline scalp electrode Fz for Patients 2–4,where KCs show the greatest amplitude, or the scalp electrode C4 for Patient 1 where placement of a midline electrode was not clinically feasible, during N2 and N3. For SEEG, only bipolar channels with average KC amplitudes greater than 100 µV were included for analysis.

**KC detection for the thalamocortical intracranial recording** was performed on a prefrontal bipolar SEEG channel estimated to lie in Brodmann's area 10 during N2 and N3 based on the multiphasic morphology outlined above.

**KC detection for EEG** was performed on the midline scalp electrode Fz, where KCs show the greatest amplitude during N2.

#### Elimination of epileptiform activity

Sleep and epilepsy can have a profound effect on one another [74] and their interactions must be considered in analyzing the intracranial recordings obtained from patients with epilepsy. The types of epilepsy which most commonly affect the graphoelements of N2 are generalized, frontal, or nocturnal frontal lobe epilepsy [74]–[76], but these were not present in the patients studied here. A concern in all patients with epilepsy is that typical KCs would be contaminated with epileptiform KCs [71] or focal epileptiform spikes in conjunction with KCs [72]. Fortunately, these are easy to detect and each KC was examined visually and all such KCs were excluded, as outlined above. In addition, we eliminated from consideration any electrode leads with consistent delta slowing, or frequent interictal spikes, or which participated in the seizure focus. The majority of the patients examined here present with temporal lobe seizures, which more often are enhanced during wakefulness as compared to sleep [75]. While these intracranial recordings must be interpreted within this clinical context, we have sought to rigorously exclude epileptic activity and to include a normal control subject's data as well.

### Computational model

#### Intrinsic currents: Thalamus

Single-compartment models of thalamocortical (TC) and reticular nucleus (RE) neurons included voltage- and calcium-dependent currents based on Hodgkin-Huxley kinetics:
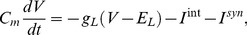
(1)with membrane capacitance C_m_ = 1 µF/cm^2^; leakage conductance g_L_ (g_L_ = 0.01 mS/cm^2^ for TC and g_L_ = 0.05 mS/cm^2^ for RE); and reversal potential E_L_ (E_L_ = −70 mV for TC and E_L_ = −77 mV for RE). I^int^ expresses a sum of active intrinsic currents and I^syn^ a sum of synaptic currents. Area of an RE cell was S_RE_ = 1.43 ·10^−4^ cm^2^ and area of a TC cell was S_TC_ = 2.9 ·10^−4^ cm^2^.

In both RE and TC, we implemented a fast sodium current, I_Na_, a fast potassium current, I_K_, a low-threshold Ca^2+^ current, I_T_, and a potassium leak current, I_KL_ = g_KL_(V-E_KL_), E_KL_ = −95 mV. TC also included a hyperpolarization-activated cation current I_h_. The expressions for voltage- and Ca^2+^-dependent transition rates for all currents are given in [16], [77]. The maximal conductances were g_K_ = 10 mS/cm^2^, g_Na_ = 90 mS/cm^2^, g_T_ = 2.2 mS/cm^2^, g_h_ = 0.017 mS/cm^2^, g_KL_ = 0–0.03 mS/cm^2^ for TC and g_K_ = 10 mS/cm^2^, g_Na_ = 100 mS/cm^2^, g_T_ = 2.3 mS/cm^2^, g_KL_ = 0.005 mS/cm^2^ for RE. In some cases, I_T_ inactivation in RE was scaled to a percentage of its original value.

#### Intrinsic currents: Cortex

Two-compartment models of cortical pyramidal (PY) neurons and interneurons (IN) included channels modeled by Hodgkin-Huxley kinetics [78]:
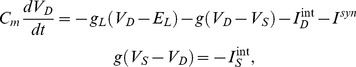
(2)with dendritic compartment membrane capacitance (C_m_) and leakage conductance (g_L_); reversal potential E_L_; membrane potentials of dendritic (V_D_) and axo-somatic compartments (V_S_); sums of active intrinsic currents in axo-somatic (I_S_^int^) and dendritic compartments (I_D_^int^); sum of synaptic currents (I^syn^); and conductance between axo-somatic and dendritic compartments g. This model was first proposed in [78] as a reduction of a multi-compartmental pyramidal cell model, based on the assumption that the current dynamics in axosomatic compartment are fast enough to ensure that *V*_S_ is always at equilibrium state, as defined by the second equation in (2). Indeed, this reduced model has relatively high Na^+^ and K^+^ conductance values (*g*_Na_ = 3000 mS/cm^2^, *g*_K_ = 200 mS/cm^2^ [78]) in the axosomatic compartment (representing axon hillock in the model). Because of the high conductance densities, smaller integration steps were needed to ensure stability of calculation when capacitance was included in the axosomatic compartment. Both models (reduced and completed) showed identical firing patterns [78].

The fast Na^+^ channels, I_Na_, were high density in the axo-somatic compartment and low density in the dendritic compartment. The axo-somatic compartment contained a fast delayed rectifier potassium K^+^ current, I_K_. The axo-somatic and dendritic compartments both included a persistent sodium current, I_Na(p)_. The dendritic compartment contained a slow voltage-dependent non-inactivating K^+^ current, I_Km_, slow Ca^2+^ dependent K^+^ current, I_K(Ca)_, high-threshold Ca^2+^ current, I_HVA_, and a potassium leak current, I_KL_ = g_KL_(V-E_KL_). See [11], [16] for the voltage- and Ca^2+^-dependent transition rates for all currents. The maximal conductances and passive properties were S_soma_ = 1.0 ·10^−6^ cm^2^, g_Na_ = 3000 mS/cm^2^, g_K_ = 200 mS/cm^2^, g_Na(p)_ = 0.07 mS/cm^2^ for axo-somatic compartment and C_m_ = 0.75 µF/cm^2^, g_L_ = 0.033 mS/cm^2^, g_KL_ = 0–0.0025 mS/cm^2^, S_dend_ = S_soma_ r, g_HVA_ = 0.01 mS/cm^2^, g_Na_ = 1.5 mS/cm^2^, g_KCa_ = 0.3 mS/cm^2^ g_Km_ = 0.01 mS/cm^2^ g_Na(p)_ = 0.07 mS/cm^2^ for dendritic compartment. E_L_ = −68 mV and E_KL_ = −95 mV. No I_Na(p)_ was modeled for IN. The resistance between compartments was R = 10 MΩ.

The firing properties in Eq. (2) depend on the ratio of dendritic area to axo-somatic area r [78] and the coupling conductance between compartments (g = 1/R). A model of a regular-spiking neuron was used for PY (r = 165) and a model of a fast spiking neuron was used for IN (r = 50).

#### Synaptic currents

All synaptic currents were calculated using:
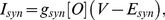
(3)with maximal conductivity g_syn_; fraction of open channels [O](t); and reversal potential E_syn_. E^syn^_AMPA_ = 0 mV for AMPA and NMDA receptors; E^syn^_GABAA_ = −70 mV for GABA_A_ receptors in RE and PY and E^syn^_GABAA_ = −80 mV for GABA_A_ receptors in TC [79]; and E^syn^_GABAB_ = −95 mV for GABA_B_ receptors. A simple phenomenological model described short-term depression of intracortical excitatory connections [11], [80]–[82]. According to this, a maximal synaptic conductance was multiplied to depression variable, *D*≤1, representing the amount of available “synaptic resources.” 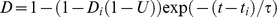, where *U* = 0.07 is the fraction of resources used per action potential, τ = 700 msec the time constant of recovery of the synaptic resources, *D*_i_ is the value of *D* immediately before the *i*_th_ event, and (*t* - *t*i) is the time after *i*_th_ event.

First-order activation schemes modeled GABA_A_, NMDA, and AMPA synaptic currents [83]. NMDA receptors' dependence on postsynaptic voltage was 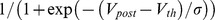
_,_ where V_th_ = −25 mV, σ = 12.5 mV [83]–[85]. A higher-order reaction scheme modeled GABA_B_ receptors, which took into account activation of K^+^ channels by G-proteins [41], [83], [86]. For all synaptic current equations see [11], [16], [77]. The maximal conductances (for each synapse) were g_AMPA(PY-PY)_ = 0.09 µS, g_NMDA(PY-PY)_ = 0.01 µS, g_AMPA(PY-TC)_ = 0.08−0.025 µS, g_AMPA(PY-RE)_ = 0.5 µS (for most simulations, but in the case of increasing this value to generate increasing number of KCs, the maximum value was 5 uS), g_AMPA(TC-PY)_ = 0.1 µS, g_AMPA(PY-IN)_ = 0.05 µS, g_NMDA(PY-IN)_ = 0.008 µS, g_GABAA(IN-PY)_ = 0.05 µS, g_AMPA(TC-IN)_ = 0.1 µS, g_GABAA(RE-RE)_ = 0.2 µS, g_GABAA(RE-TC)_ = 0.2 µS, g_GABAB(RE-TC)_ = 0.04 µS, g_AMPA(TC-RE)_ = 0.4 µS.

Spontaneous miniature IPSPs and EPSPs on the cortical synapses followed the same equations as the regular PSPs. Poisson processes [87] modeled their arrival times, with time-dependent mean rate 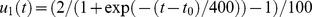 or 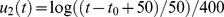, where t_0_ is a time instant of the last presynaptic spike [11]. The mini amplitude was ∼0.75 mV.

#### Network geometry and stimulation

The simulated geometry was a two layer network representing the cortex and the thalamus (Figure 3A). Each layer was one-dimensional. The cortical layer consisted of 100 PY neurons and 25 INs. The thalamic layer consisted of 50 RE neurons and 50 TC neurons. The fan-out from PY to PY was radius 5 for AMPA and NMDA connections, radius 1 for AMPA and NMDA PY to IN synapses, radius 5 for GABA_A_ IN to PY synapses, radius 10 for AMPA TC to PY, radius 2 for AMPA TC to IN synapses, radius 5 for AMPA PY to TC and PY to RE, and radius 5 for AMPA and GABA_A_ synapses between TC and RE neurons (Figure 3A & B). In some simulations, a subset of PY neurons projected to all RE neurons, while the remaining PY neurons maintained their original projections to 5 RE neurons each (Figure 3C). Depolarization was applied to all RE neurons in the original configuration (Figure 3B) and to the subset of PY neurons projecting to all RE neurons in the reconfiguration (Figure 3C). GABAergic IN-IN synapses were not included. Some of the intrinsic parameters of the neurons in the network were initialized with random variability (Gaussian distribution with σ = 5–10%) to insure the robustness of the results [77]. Neurons were stimulated by injecting currents into RE (14 to 915pA) or PY (0.5 to 80pA) neurons. Spontaneously occurring KCs were produced without the addition of these currents. Most simulations ran for 10 s, while simulations of spontaneous activity ran for 200 s.

### Measures of Model Activity

**The average membrane potential** for each population was calculated by averaging over each cell's membrane potential; application of a −50 mv spiking threshold prevented the signal from being contaminated by spiking activity. This was taken as an approximation of the LFP for this population [88].

**Firing rate** was calculated using the membrane potentials from each cell. The firing rate for an individual cell was calculated over a 5 ms or 100 ms bin: a spike was counted each time a cell's membrane potential reached above −20 mv and then dropped below −30 mv the following millisecond. Firing rate for a population was calculated in spikes per second by multiplying the number of spikes in a bin by the number of bins in 1 sec (e.g. 5 ms = 20bins/sec) and dividing by the number of neurons in the population (e.g. 100 for PY neurons).

**Population specific measures** of conductance, current, calcium levels, and inactivation and activation states were measured for PY, TC, and RE neurons. For PY neurons, these measures were: average axosomal I_Na(p)_; average dendritic I_Na(p)_; average I_K(Ca)_; average I_KL_; and level of calcium. For TC neurons, these were: average g_h_ conductance; average I_h_; average I_T_; average I_KL_; I_T_ inactivation and activation states; sum of PY to TC total currents; and level of calcium. For RE neurons, these were: average I_T_; average I_K_ current; I_T_ inactivation and activation states; and sum of PY to RE total currents.

**Spindle and High Gamma Power** were calculated using Morlet wavelets or the Hilbert transform with customized Matlab routines incorporating the publicly available FieldTrip toolbox [89]. For modeling results, spindling power was calculated from 8–13 Hz (the standard deviation of the wavelet was 2 Hz in the frequency domain and 80 ms in the time domain), while HGP was calculated from 70–200 Hz (the standard deviation of the wavelet was 10 Hz in the frequency domain and 16 ms in the time domain). Although the spindle band for this analysis is lower than is typically observed in scalp EEG, it corresponds to the frequencies present in the spindles generated by the model. Power was calculated for individual neurons and trials and then averaged. No distinction was made in the model corresponding to the distinction between frontal maximum slow spindles and parietal maximum fast spindles seen in scalp EEG, because it is not clear what their intracortical basis is, and their modeling would require more neurons than are currently practical to compute.

For thalamocortical SEEG data, the cortical bipolar channel was band pass filtered to between 60–120 Hz (to measure high gamma) and the thalamic bipolar channel was band passed filtered to between 12–16 Hz (to measure spindling). Because the normal spindle frequency in the human thalamus is unknown, the spindle frequency band for the thalamus used for analysis was chosen based on the dominant frequencies present in spontaneous spindles in these recordings. The data were epoched using cortical KC times and the Hilbert transform was applied to each KC. The absolute value of the Hilbert was averaged over all KCs for the cortical (for high gamma) and thalamic (for spindling) channels. Time frequency analysis (5–120 Hz) of thalamic data was performed using EEGLAB [90], with the most negative peak of the KC at time zero using −1.5 to −0.5 seconds as a comparison baseline at a p<0.01 threshold.

**Baseline corrected values** were calculated when multiple simulations evoking KCs were run with the same input values. In these cases, the same random seed was used for an evoked KC run and a no stimulation run. Both runs used the same parameters, except that the evoked KC run either applied PY stimulation or RE depolarization. Each evoked KC run was baseline corrected using its corresponding no stimulation run. At a time point of interest, the baseline corrected value was calculated by subtracting the value in the no stimulation condition, B, from the corresponding value in the evoked condition, A. The calculation for a percent change was: ((A–B)/B)*100.

**Cortical KCs** were identified in PY neurons by their hyperpolarized membrane potential, decreased membrane potential fluctuations (as indicated by HGP), and decreased firing rate (less than 10 spikes per 100 ms, for at least 200 ms).
